# Cyclic Strain and Macrophage‐Mediated Transport Govern Micron‐Sized PM_2_._5_ Translocation across the Air–Blood Barrier

**DOI:** 10.1002/advs.202511955

**Published:** 2025-10-29

**Authors:** Yongjian Li, Jinlong Xu, Zujie Gao, Haosheng Chen

**Affiliations:** ^1^ Department of Mechanical Engineering Tsinghua University Beijing 100084 P. R. China; ^2^ State Key Laboratory of Tribology in Advanced Equipment Tsinghua University Beijing 100084 P. R. China

**Keywords:** air–blood barrier, cyclic strain, macrophage, PM_2_._5_, translocation

## Abstract

PM_2_._5_ translocated across the air‐blood barrier (ABB) can accumulate in the lungs and extrapulmonary organs, leading to both pulmonary and systemic pathophysiological effects. However, the mechanisms governing the translocation of micron‐sized PM_2_._5_ (MPs) remain unclear. Here, a physiologically relevant in vitro ABB model is developed, comprising a triple co‐culture system under respiratory‐like cyclic strain. Using this platform, it is demonstrated that cyclic stretching enhances dTHP‐1 phagocytosis and transmigration, synergistically promoting macrophage‐mediated MPs translocation across the ABB in combination with strain‐induced disruption of epithelial tight junctions. This cooperative mechanism governs MPs translocation across the ABB and markedly increases the transport of 2 µm particles compared to static conditions. By using scavenger receptor A (SR‐A) inhibitors, this process are confirmed to be predominantly driven by macrophage phagocytosis. Furthermore, this study explores the potential fate of particles post‐translocation, proposing their subsequent release from macrophages via active lysosomal exocytosis and passive release mediated by cell death. These findings provide new insights into mitigating PM_2_._5_‐induced health risks and inform macrophage‐assisted pulmonary drug delivery strategies.

## Graphical abstract

Using a dynamic, tri‐culture air‐blood barrier model, this study reveals that macrophage‐mediated transport governs micron‐sized particle translocation under cyclic strain. The process is highly size‐dependent that 2 µm particles are transported by macrophages, 0.2 µm particles diffuse passively, and 5 µm particles are effectively blocked. After crossing the barrier, particles are released from macrophages via lysosomal exocytosis or cell death.

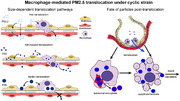

## 1 Introduction

Air pollution causes over two million deaths annually,^[^ ^1^ ^]^ representing a major public health challenge.^[^ ^2^, ^3^ ^]^ Fine particulate matter (PM_2_._5_) is especially hazardous due to its small size and large surface area, enabling deep lung penetration and delivery of toxic substances.^[^ ^4^, ^5^ ^]^ Notably, the PM_2_._5_ classification encompasses a broad range of particle sizes, including both nanoparticles and larger, micron‐sized particles (MPs, diameter ≥1 µm), which may carry a higher load of adsorbed toxic substances due to their greater volume.^[^ ^6^ ^]^ The health risks of MPs have not received sufficient attention. Their deposition pattern within the respiratory tract further amplifies this risk, as particles between 1 and 5 µm tend to deposit in the tracheobronchial and alveolar regions, strongly provoking local inflammation and oxidative stress.^[^ ^7^ ^]^ Short‐term exposure to high concentrations of MPs can trigger an acute inflammatory response,^[^ ^8^ ^]^ exacerbate respiratory diseases such as asthma,^[^ ^9^ ^]^ and increase the risk of cardiovascular events and overall mortality.^[^ ^10^ ^]^ Long‐term exposure causes persistent damage, contributing to the development of chronic respiratory diseases and impaired lung function, such as increased COPD risk, and is closely associated with childhood asthma and age‐related lung function decline.^[^ ^11^ ^]^ In addition, the International Agency for Research on Cancer (IARC) has classified airborne particulate matter, of which MPs are a major component, as a Group 1 carcinogen, confirming its causal link to lung cancer.^[^ ^12^ ^]^ More alarmingly, studies have shown that PM_2_._5_, including MPs, can cross the air–blood barrier (ABB) and enter the circulation, leading to endothelial dysfunction, vascular inflammation, and increased cardiovascular risk.^[^ ^13^ ^]^ Moreover, PM_2_._5_ can accumulate in organs such as the brain,^[^ ^14^ ^]^ kidneys,^[^ ^15^ ^]^ and liver,^[^ ^16^ ^]^ thereby contributing to systemic diseases.^[^ ^17^ ^]^ Despite these risks, the precise mechanisms by which larger MPs breach the ABB and induce systemic effects remain poorly understood, representing a critical gap in current knowledge. Conversely, the characteristics of MPs, such as their large drug loading capacity, also make them promising for macrophage‐mediated pulmonary drug delivery.^[^ ^18^ ^]^ Therefore, investigating the translocation mechanisms of MPs is essential for evaluating their health risks, guiding protective strategies, and informing the development of new therapies. A deeper mechanistic understanding is required to connect epidemiological evidence linking particle exposure to systemic diseases with cellular‐level events at the ABB, and such insights may also reveal the potential of endogenous immune cells to serve as carriers for targeted drug delivery, a concept that is attracting growing attention in advanced therapeutics.

Particle size plays a critical role in the translocation of PM across the ABB, yet the underlying mechanisms vary substantially with particle size.^[^ ^19^, ^20^ ^]^ For nanoparticles (NPs), multiple pathways for translocation have been identified, including paracellular diffusion,^[^ ^21^ ^]^ transcellular transport via distinct endocytic mechanisms,^[^ ^22^ ^]^ and indirect translocation facilitated by membrane transporters.^[^ ^23^ ^]^ The efficiency of these pathways is strongly influenced by particle properties such as size, charge, and surface chemistry, and generally decreases with increasing particle size.^[^ ^24^, ^25^, ^26^ ^]^ However, increasing in vivo evidence indicates that MPs can traverse the ABB and enter the systemic circulation, yet the mechanisms established for NPs fail to account for MPs behavior. Owing to their larger size, MPs are excluded from paracellular routes, and limited epithelial uptake often results in intracellular sequestration rather than translocation across the epithelial–endothelial barrier.^[^ ^27^, ^28^, ^29^ ^]^ This mechanistic gap suggests that MP translocation is governed by distinct biological processes involving complex cellular interactions beyond those identified for NPs.^[^ ^30^ ^]^

To elucidate the mechanisms by which PM translocate across the air–blood barrier (ABB), a variety of biological models have been developed.^[^ ^31^ ^]^ However, animal models do not allow direct in situ observation of microscopic processes, and the complexity of the in vivo environment limits mechanistic investigations. Inspired by the pioneering work of Huh et al.,^[^ ^32^ ^]^ lung‐on‐a‐chip (LoC) devices incorporating an epithelial–endothelial bilayer and subjected to cyclic mechanical strain have attracted considerable attention and are regarded as the most physiologically relevant dynamic models of the alveolar barrier. It is generally accepted that LoCs should reproduce cyclic stretching of ≈0.2 Hz and 5–12% linear strain to mimic the breathing process,^[^ ^33^ ^]^ with designs evolving from in‐plane to more physiologically relevant out‐of‐plane modes. Although such dynamic models have been successfully applied to study drug responses,^[^ ^34^ ^]^ PM toxicity,^[^ ^35^ ^]^ and viral infections,^[^ ^36^ ^]^ they remain limited to epithelial–endothelial co‐cultures and thus fail to explain the translocation of larger PM_2_._5_ particles.

Our previous in vivo imaging work demonstrated that 2 µm polystyrene microspheres gradually entered the circulation and accumulated in distal organs, directly confirming that large particles can cross the ABB.^[^ ^37^ ^]^ Yet conventional static models impose severe restrictions^[^ ^38^ ^]^ that only a small fraction of 1 µm particles can traverse the epithelial layer,^[^ ^39^ ^]^ and 5 µm particles are almost completely blocked.^[^ ^40^ ^]^ By contrast, dynamic stretching models show that cyclic strain increases epithelial permeability and facilitates paracellular routes, thereby enhancing particle transport.^[^ ^41^ ^]^ Nonetheless, the limited efficiency observed in these models suggests the involvement of additional biomechanical and cellular processes.

For large PM_2_._5_ particles, immune cells such as alveolar macrophages (AMs) are thought to play a decisive role through phagocytosis and active transport.^[^ ^42^, ^43^ ^]^ AMs display robust phagocytic activity, particularly for particles in the 1–10 µm range, with peak efficiency ≈1 µm.^[^ ^44^, ^45^ ^]^ Studies further show that particles as small as 20 and 200 nm can also be internalized by AMs and translocated across barriers.^[^ ^46^ ^]^ Moreover, using macrophages as carriers is a proven strategy for targeted drug delivery, enabling nanoparticle transit across the blood‐brain barrier,^[^ ^47^ ^]^ and their accumulation within the tumor microenvironment.^[^ ^48^ ^]^ The absence of biomechanical coupling in previous models may account for their limited ability to replicate observed MP translocation and underscores a critical gap in our current understanding of its underlying mechanisms.^[^ ^49^, ^50^, ^51^ ^]^ However, most studies introducing immune cells into barrier systems remain confined to static Transwell configurations, lacking the dynamic stretching necessary to capture the regulation of macrophage migration, phagocytosis, and translocation.

To address this gap, we developed a physiologically relevant LoC model integrating epithelial, endothelial, and macrophage co‐cultures under cyclic mechanical strain. The core strength of this system lies in its functional integration, as it reveals for the first time a strong synergistic effect between strain‐induced epithelial disruption and macrophage‐mediated transport, which together greatly enhance the efficiency of microparticle translocation. This discovery fills the missing link in current dynamic models by establishing a direct biomechanical‐immune coupling mechanism, providing not only a powerful in vitro platform for assessing the health risks of PM_2_._5_ but also a promising strategy for macrophage‐based pulmonary drug delivery.

To address this gap, we developed a physiologically relevant LoC model that integrates epithelial, endothelial, and macrophage co‐cultures under cyclic mechanical strain to investigate the translocation of micron‐sized PM_2_._5_. Our results demonstrate that transport is jointly driven by strain‐induced disruption of epithelial junctions and macrophage‐mediated phagocytosis. Pharmacological inhibition experiments confirmed that this translocation is a phagocytosis‐dependent process. This synergistic effect markedly enhances the translocation efficiency of 2 µm particles and exhibits clear size dependence. Furthermore, this study provides initial insights into the downstream fate of these particles, exploring their subsequent release from macrophages via both active exocytosis and passive, cell‐death mediated pathways. By elucidating the full pathway from uptake to translocation and subsequent release, this work not only reveals the amplifying interplay between mechanical strain and immune cell transport, thereby filling a critical gap in current dynamic models by establishing a biomechanical–immune coupling mechanism, but also provides a novel in vitro platform and research avenue for both PM_2_._5_ health risk assessment and macrophage‐based pulmonary drug delivery strategies.

## 2 Results

### 2.1 Construction of In Vitro ABB Model for PS2 Translocation Mechanism Analysis

**Figure 1:**
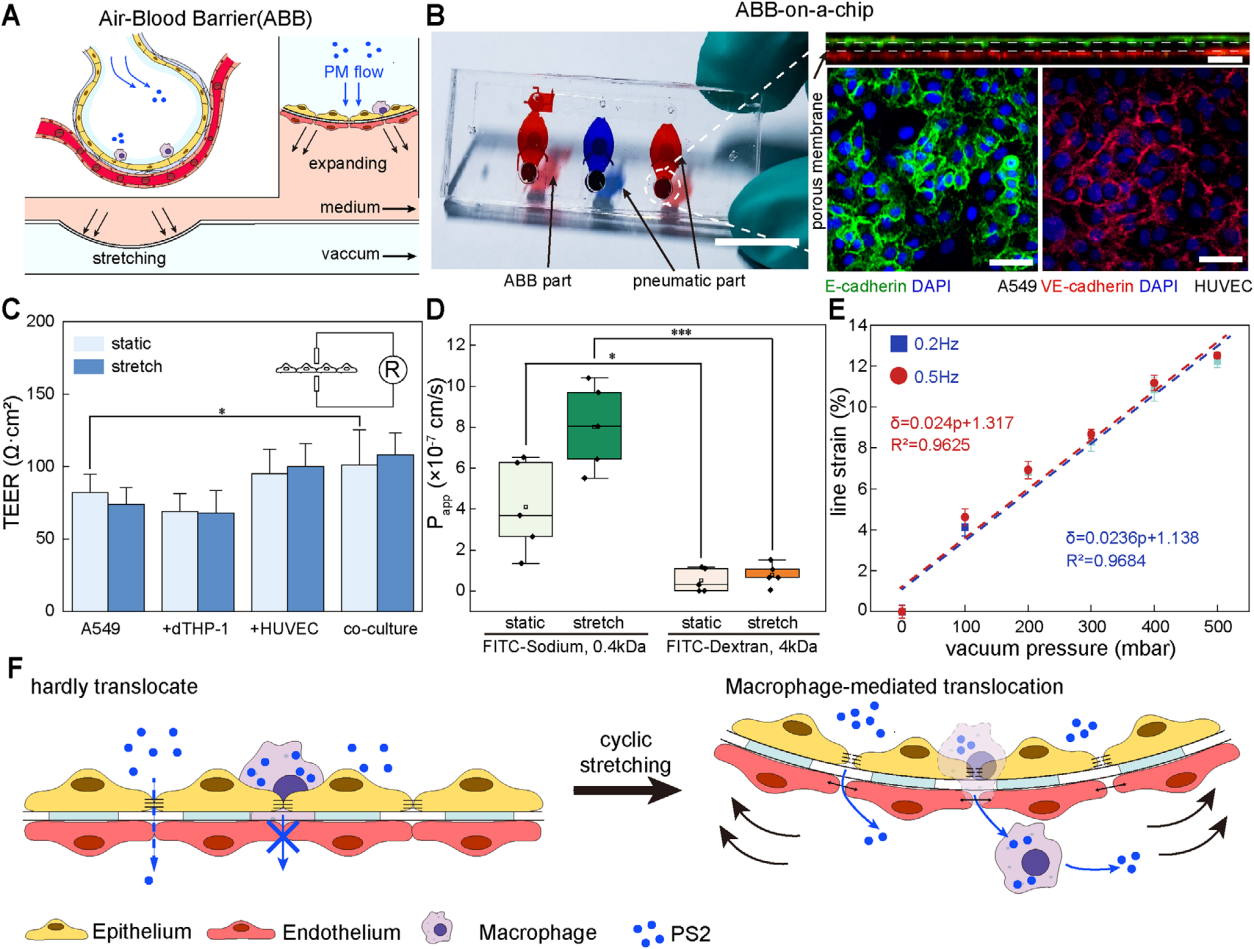
Fabrication of the in vitro ABB model and the synergistic mechanism of PM_2_._5_ translocation via cyclic strain and macrophage mediation. A) Schematic of the ABB model illustrating diaphragm‐like cyclic strain generated via vacuum actuation. B) Photograph of the ABB chip with parallel units (scale bar, 1 cm), alongside confocal images showing A549 and HUVEC layers stained for E‐cadherin (green) and VE‐cadherin (red) (scale bar, 50 µm). C) TEER measurements of different culture configurations under static or 5% cyclic strain (*n*  =  6 independent chip chambers; mean ± s.d.; ^*^ *p*< 0.05). D) Translocation efficiency of FITC–sodium and FITC–dextran under both static and stretched conditions (*n*  =  5 independent chip chambers; mean ± s.d.; ^*^ *p* < 0.05, ^***^ *p* < 0.001). E) Linear correlation between vacuum pressure and membrane strain amplitude at 0.2 and 0.5 Hz. F) Proposed mechanism of PS2 translocation mediated by cyclic stretching and macrophage involvement.

To mimic the physiological microenvironment of the alveolars, we established a biomimetic on‐chip ABB model with controllable cyclic strain and multiple parallel chambers for direct apical exposure to PM_2_._5_ suspensions or aerosols.^[^ ^32^, ^52^ ^]^ Negative pressure applied beneath the ABB chamber deforms a PDMS membrane, stretching the tri‐culture layer to mimic alveolar expansion (**Figure** **1**A).^[^ ^33^ ^]^ The amplitude and waveform of strain were precisely controlled using a programmable pressure controller (OB1+ MK3, Elveflow, France), enabling reproduction of both physiological (5–12% strain) and pathological breathing patterns. The ABB chamber contained a flexible, porous PDMS membrane (20 µm thick, 8 µm pore diameter), which provided structural support and permitted interaction between apical and basal cell layers. At 12 h after seeding, epithelial and endothelial cells formed confluent and compact monolayers, and a dual co‐culture was established in the ABB region (Figure 1B). Immunofluorescence imaging confirmed E‐cadherin and VE‐cadherin expression at cell junctions, indicating intact epithelial (A549) and endothelial (HUVEC) layers.^[^ ^53^ ^]^

To assess the physiological relevance of the model, we first evaluated barrier integrity and solute permeability.^[^ ^54^ ^]^ The triple co‐culture configuration produced the highest transepithelial electrical resistance (TEER), reflecting stable cell–cell junctions (Figure 1C). TEER values remained consistent under 5% cyclic strain compared with static culture, confirming that short‐term deformation did not affect overall barrier function. To evaluate the integrity and size‐selective function of the barrier, solute permeability was assessed using sodium fluorescein (376 Da) and FITC‐dextran (4 kDa) as model hydrophilic tracers. The barrier demonstrated clear size selectivity, with the apparent permeability coefficient (Papp) for sodium fluorescein (8.02±2.08 × 10^−^⁷ cm/s, *n* = 5 independent chambers, mean±S.D.) being significantly higher than that for the larger 4 kDa FITC‐dextran (0.79±0.54 × 10^−^⁷ cm/s, *n* = 5 independent chambers, mean ± S.D.,), confirming the formation of a functional and selective barrier (Figure 1D). This ability to differentiate solutes by size suggests the model is also suitable for assessing particulate translocation, a conclusion supported by comparison with similar in vitro barrier systems (Table S1, Supporting Information). The strain of stretching increased linearly with pressure up to 15%, enabling precise control of deformation (Figure 1E). Comparable strain profiles were observed at 0.2 and 0.5 Hz, suggesting that breathing frequency had no measurable effect on strain behavior within this range.

Using this platform, we proposed a mechanism in which large PM_2_._5_ particles cross the ABB via macrophage‐mediated uptake under cyclic mechanical strain (Figure 1F). Stretching disrupted epithelial tight junctions and widened intercellular gaps, allowing particle and cell passage. Macrophages on the apical side internalized particles and mediated their transport across the barrier. These observations led us to further investigate how mechanical strain modulates particle translocation across the ABB.

### 2.2 Effect of Cyclic Mechanical Strain on PM_2_._5_ Translocation across the ABB

Previous studies have shown that cyclic mechanical strain can disrupt epithelial architecture by altering cytoskeletal organization and intercellular junctions.^[^ ^55^ ^]^ To investigate this effect in our ABB model, A549 monolayers cultured on porous membranes were fixed and stained for nuclei and F‐actin after 5 min of strain application (0%, 5%, 15%). Our selected strain parameters were designed to simulate the mechanical environment of the lung under different physiological and pathological states. A cyclic strain of 5% represents the physiological baseline deformation experienced by alveoli during resting breathing (tidal breathing) in healthy individuals.^[^ ^56^, ^57^ ^]^ In contrast, the 15% strain amplitude simulates the pathological overstretch that some alveolar regions may endure under conditions such as acute respiratory distress syndrome (ARDS), and this high‐magnitude stress is a key factor in ventilator‐induced lung injury (VILI).^[^ ^58^ ^]^ The duration of strain application was chosen to consider the combined effects of mechanical stress and particle exposure. The short 5‐min timeframe was primarily used to capture the rapid, dynamic cellular responses to mechanical stimulation, such as the immediate remodeling of the F‐actin cytoskeleton. The 2–4 h exposure duration, on the other hand, is a widely adopted time point in in vitro toxicology studies, sufficient for observing subsequent key biological events induced by particulate matter.^[^ ^59^ ^]^ During this period, it is possible to assess the disruption of barrier integrity caused by the synergistic action of mechanical forces and particles, including the decreased expression of tight junction proteins, while also providing ample time for macrophages to complete the recognition, phagocytosis, and initiation of transmigration of micron‐sized particles.

**Figure 2:**
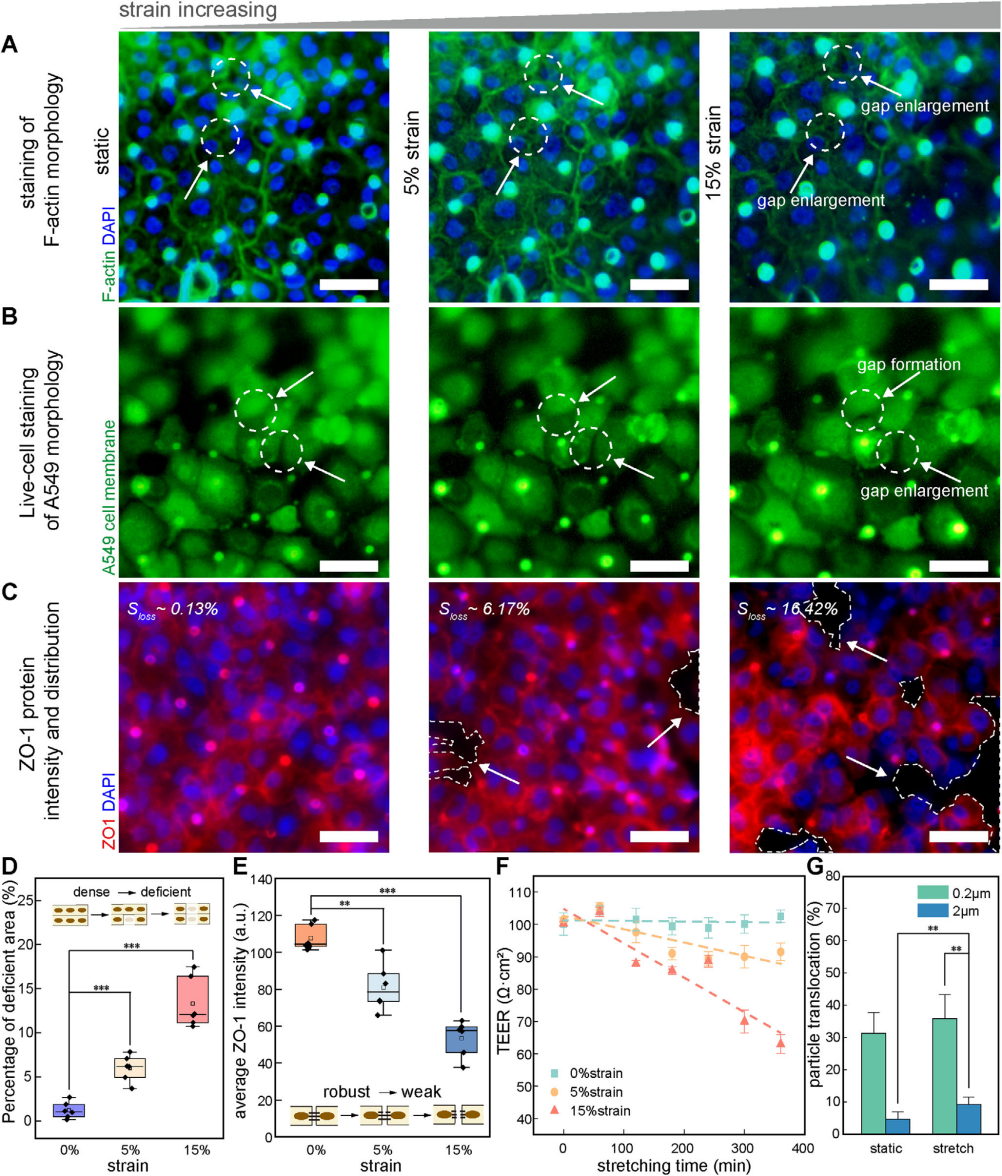
Cyclic mechanical strain disrupts epithelial junctions and promotes PS2 translocation across the ABB. A) Representative immunofluorescence images of fixed A549 cells showing F‐actin (green) and nuclei (blue) after 5 min of cyclic strain at 0%, 5%, and 15%. Dashed circles and arrows indicate intercellular gap widening (scale bar, 50 µm). B) Live‐cell membrane imaging of A549 cells under identical strain conditions showing cell boundary separation and deformation (scale bar, 50 µm). C) ZO‐1 (red) and nuclei (blue) staining after 2 h of cyclic strain, revealing progressive loss of ZO‐1 signal and junction integrity with increasing strain (scale bar, 50 µm). D) Quantification of junctional discontinuities, showing the percentage of deficient area (voids) in the monolayer increases with strain (*n* = 6 independent fields of view; mean ± s.d.; ^***^ *p*< 0.001). E) Quantification of average ZO‐1 intensity under different strain levels (*n* = 6 independent fields of view; mean ± s.d.; ^**^ *p* < 0.01, ^***^ *p* < 0.001). F) Time‐course TEER measurements under different strain levels, with linear fits showing strain‐dependent decline (*n* = 6 independent chambers; mean ± s.d.). G) Translocation percentages of PS0.2 and PS2 after 2 h under static or 5% strain (*n* = 6 independent chambers; mean ± s.d.; ^**^ *p* < 0.01).

Increasing strain induced actin remodeling and enlargement of cell–cell gaps (**Figure** **2**A). Strain magnitude was calibrated using pore spacing on blank membranes (Figure S1, Supporting Information). Live‐cell imaging revealed intercellular detachment and membrane deformation under strain (Figure 2B). After 2 h of stretching, immunostaining showed reduced ZO‐1 localization at junctions, along with increased intercellular spacing and disrupted epithelial morphology (Figure 2C). Moreover, these structural changes became prominent under 15% strain, with an evident loss of junctional continuity. Quantitative analysis confirmed this strain‐dependent disruption. Specifically, the percentage of deficient area (voids) within the monolayer showed a significant, dose‐dependent increase from 1.21 ± 0.94% (*n* = 6 independent fields of view; mean ± s.d.) at 0% strain to 5.98 ± 1.49% at 5% strain, and further to 13.32 ± 2.88% at 15% strain (*n* = 6 independent fields of view; Figure 2D), providing direct evidence of physical barrier failure. This was supported by a corresponding decline in the average ZO‐1 intensity at the remaining junctions, which decreased from 107.72 ± 6.86 at 0% strain to 80.97 ± 12.63 at 5% strain and 53.51 ± 9.73 at 15% strain (n = 6 independent fields of view; Figure 2E).

To further evaluate the relative contributions of particle exposure and mechanical strain, we compared their individual effects on ABB integrity. In our model, PS particles alone caused minimal epithelial disruption, whereas cyclic strain induced marked intercellular gap expansion and reduced ZO‐1 expression. Consistently, TEER measurements showed a significant decrease following prolonged high‐amplitude strain, indicating barrier impairment (Figure 2F; *n* = 6 independent chip chambers). To assess size‐dependent translocation, PS0.2 and PS2 particles (20 µg mL^−1^) were applied under static or 5% strain for 2 h. PS0.2 particles crossed the barrier under both conditions, while PS2 particles exhibited translocation only with cyclic strain, indicating a size‐dependent translocation pattern in which PS2 requires strain‐induced junctional remodeling to cross the ABB (Figure 2G; *n* = 6 independent chip chambers).

### 2.3 dTHP‐1–Mediated Translocation of PS2 Particles across the ABB under Cyclic Strain

While mechanical strain facilitates junctional remodeling, cellular mechanisms such as phagocytosis may also contribute to particle translocation across the ABB. To investigate the role of macrophages in PM_2_._5_ translocation, we used PMA‐differentiated THP‐1 (dTHP‐1) cells to model alveolar macrophages.^[^ ^60^ ^]^ dTHP‐1 cells exhibited strong phagocytic activity, whereas undifferentiated THP‐1 cells showed low uptake. Particle size had little effect on total uptake, consistent with previous reports that macrophages internalize particles based on volume (Figure S3, Supporting Information). After integration into the ABB model, dTHP‐1 cells retained this phagocytic capacity, as evidenced by intracellular localization of fluorescent PS particles (Figure S4, Supporting Information).

**Figure 3:**
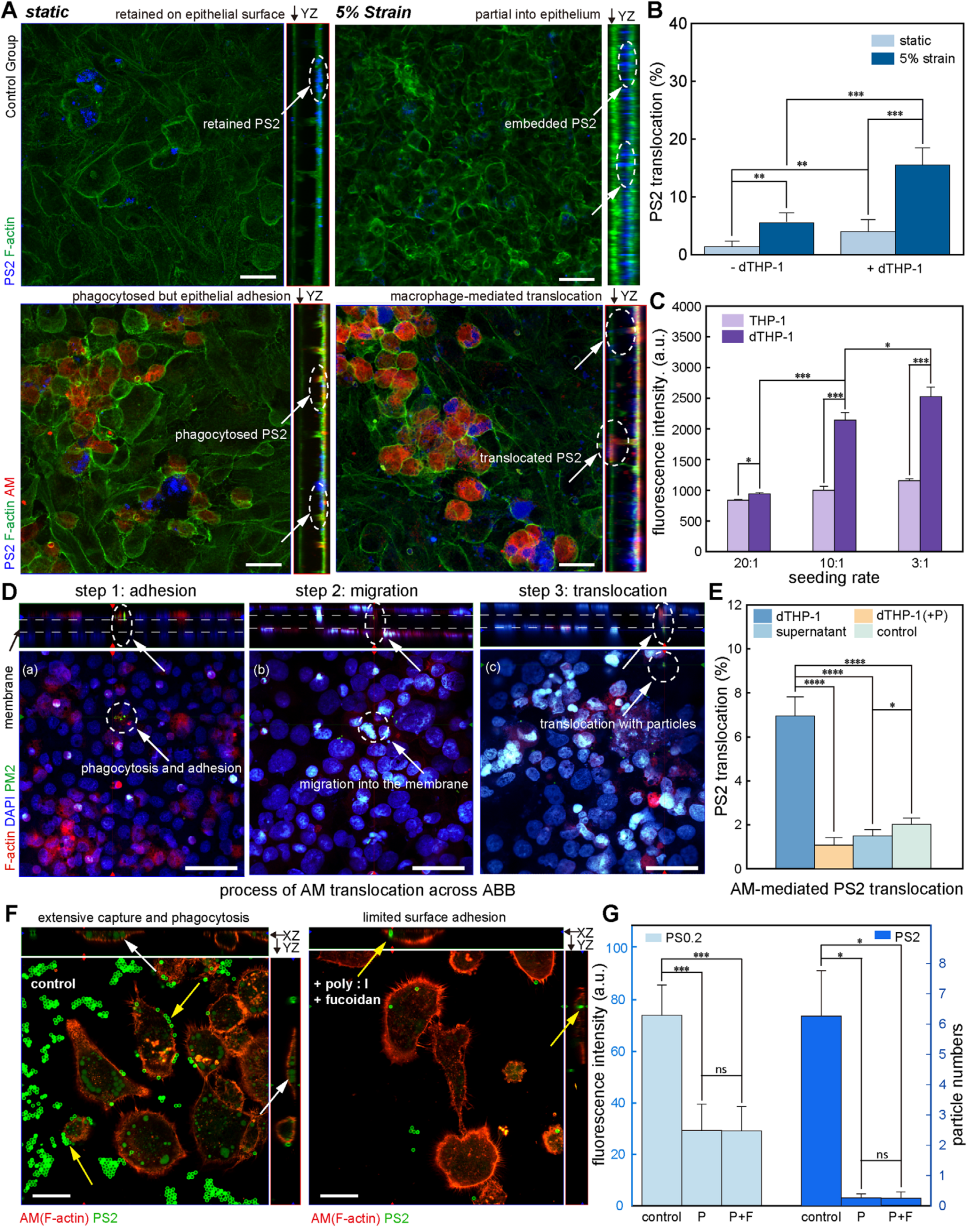
dTHP‐1–mediated translocation of PS2 particles across the ABB under cyclic strain. A) CLSM images showing PS2 particle localization (blue) at the ABB under static and 5% cyclic strain conditions, with or without dTHP‐1 cells. F‐actin (green) and macrophage membranes (red) are labeled to visualize cell architecture. Orthogonal YZ projections indicate PS2 translocation across the membrane (scale bar, 50 µm). B) Quantification of PS2 translocation (%) in the presence or absence of dTHP‐1 cells under static or strain conditions (*n* = 6 independent chambers; mean ± s.d.; ^**^ *p *< 0.01, ^***^ *p *< 0.01). C) Fluorescence intensity of PS2 on the basal side at varying dTHP‐1 seeding ratios (20:1, 10:1, 3:1), indicating a dose‐dependent increase (*n* = 6 independent chambers; mean ± s.d.; ^*^ *p *< 0.05, ^***^ *p *< 0.01). D) Representative images showing the sequential steps of dTHP‐1–mediated PS2 translocation across the ABB: (a) adhesion, (b) migration, and (c) translocation. F‐actin (red), PS2 (green), and nuclei (blue) are shown (scale bar, 50 µm). E) PS2 translocation rates under three conditions: with dTHP‐1 cells, with dTHP‐1 cells treated with poly(I) and fucoidan, with dTHP‐1 supernatant, and without dTHP‐1 (control) (*n* = 6 independent chambers; mean ± s.d.; ^*^ *p *< 0.05, ^****^ *p *< 0.0001). F) Representative confocal images of dTHP‐1 cells (stained for F‐actin in red) showing phagocytosis of PS2 (green) under control conditions (left) and after treatment with poly(I) and fucoidan (right). White arrows indicate internalized particles, and yellow arrows indicate particles adhered to the cell surface (scale bar, 20 µm). G) Quantitative analysis of particle phagocytosis by dTHP‐1 cells (*n* = 3 independent fields of view; mean ± s.d.; ^*^ *p *< 0.05, ^***^ *p *< 0.001, ns means no significance). For 0.2 µm particles (PS0.2), uptake was measured by mean fluorescence intensity per cell (left axis). For 2 µm particles (PS2), uptake was measured by the average number of internalized particles per cell (right axis).

Using the ABB platform, PS_2_ translocation was assessed under various experimental conditions. Confocal imaging revealed minimal basal particle distribution under static conditions without macrophages (**Figure** **3**A). The presence of dTHP‐1 cells slightly increased basal fluorescence, with limited particle penetration indicated by white arrows. Under 5% cyclic strain alone, PS_2_ accumulated along the basement membrane. In contrast, combined application of dTHP‐1 cells and cyclic strain led to pronounced translocation, with red‐labeled macrophages and blue particles colocalized at the basal side (dashed circles). PS2 translocation increased from 1.5 ± 0.7% (no macrophages, static) to 3.9 ± 2.2% (dTHP‐1, static), and further to 5.2 ± 2.3% and 15.5 ± 3.0% under cyclic strain without and with dTHP‐1 cells, respectively (Figure 3B). These results demonstrate that cyclic deformation promotes particle translocation, with a synergistic effect observed in the presence of macrophages. We increased the seeding ratio of dTHP‐1 cells from 20:1 to 3:1, which significantly enhanced PS2 translocation, but further increases produced no additional effect, suggesting saturation of the transport mechanism due to physical or functional constraints of the ABB (Figure 3C).

We further confirmed that PM_2_._5_ translocation across the barrier is mediated by differentiated macrophages through phagocytosis. Under low‐intensity fluorescence staining, PS2 particles were observed inside dTHP‐1 cells that migrated across the ABB. Red‐stained F‐actin outlines the epithelial structure, and blue‐stained nuclei indicate cell positions. White arrows mark sequential stages of dTHP‐1–mediated translocation: a) phagocytosis of PS2 at the apical surface, b) vertical migration into the porous membrane, and c) delivery to the basal side. This process illustrates how macrophages transmigrate the epithelial layer and transport internalized particles across the ABB (Figure 3D). To directly visualize the process of macrophage‐mediated transport, time‐lapse CLSM imaging was utilized to monitor the dynamics of individual dTHP‐1 cells. The resulting images captured a representative macrophage (red) with a 2 µm PS particle (blue) clearly internalized and co‐localized within its cytoplasm. This particle‐laden macrophage was observed actively migrating from the apical surface of the epithelial monolayer (green) and penetrating downward into the cell barrier. This dynamic translocation process is illustrated in a representative time‐lapse series (Figure S5, Supporting Information) and a more extensive recording (Video S2, Supporting Information).

Moreover, the effect of cyclic mechanical strain on macrophage migration was further assessed under PS particle exposure.^[^ ^61^, ^62^ ^]^ Under static conditions, most dTHP‐1 cells remained on the epithelial surface, with few reaching the basement membrane (Figure S6, Supporting Information). In contrast, cyclic strain increased the number of macrophages penetrating the porous membrane (white arrows). Concurrently, epithelial cells became more elongated and rhomboidal in shape. To determine whether these changes were dependent on particle stimulation, dTHP‐1 cells were introduced without PS particles. Macrophage adhesion to the epithelial layer was reduced, and translocation was rarely observed (Figure S7, Supporting Information). These findings indicate that the ABB microenvironment remains stable under physiological strain, while particle exposure is required to activate macrophage migration and translocation.

To further elucidate whether PS2 translocation is driven by active macrophage phagocytosis, rather than by simple surface adhesion or soluble factors, a series of validation experiments was conducted. First, we separated the cell suspension and the particle‐containing supernatant after co‐incubating dTHP‐1 cells with PS2 and introduced them independently into the ABB model. A significant increase in particle translocation was observed only in the group containing cells, a finding supported by fluorescence imaging that revealed dense, colocalized particle clusters on the basal side only in the presence of macrophages (Figure S8, Supporting Information). As previous studies have suggested that macrophages recognize and internalize micron‐sized particles via scavenger receptors such as SR‐A and MARCO,^[^ ^63^, ^64^ ^]^ we hypothesized that PS2 translocation is dependent on phagocytosis mediated by such receptors. To test this hypothesis, we treated the cells with two broad‐spectrum competitive inhibitors of scavenger receptors, poly(I) and fucoidan.^[^ ^65^ ^]^ The results showed that the addition of these inhibitors significantly reduced the level of macrophage‐mediated PS2 translocation to a level comparable to the control group without macrophages (Figure 3E). Interestingly, under the same particle concentration, the control group also appeared to retain more extracellular particles, potentially due to enhanced cell adhesion.

We then directly visualized the effect of the inhibitors on the phagocytosis step using a separate in vitro assay, staining for F‐actin to determine the particle‐membrane relationship. CLSM images clearly revealed that, compared to the numerous particles internalized in the control group, the inhibitor treatment significantly reduced the number of PS2 particles bound to the cell surface and within the cytoplasm (Figure 3F). Quantitative analysis of phagocytosis further confirmed this, showing that the inhibitor treatment drastically decreased the average number of PS2 particles phagocytosed by dTHP‐1 cells. A certain degree of inhibition was also observed for PS0.2 particles, which may be attributed to a reduced particle capture efficiency (Figure 3G). The clear extension of the actin cytoskeleton confirms that the inhibitors did not significantly affect cell migration or motility. All these results demonstrate that PS2 translocation across the barrier is a process predominantly driven by scavenger receptor‐mediated phagocytosis in macrophages.

### 2.4 Size‐Dependent Translocation Mechanisms of PM across the ABB

**Figure 4:**
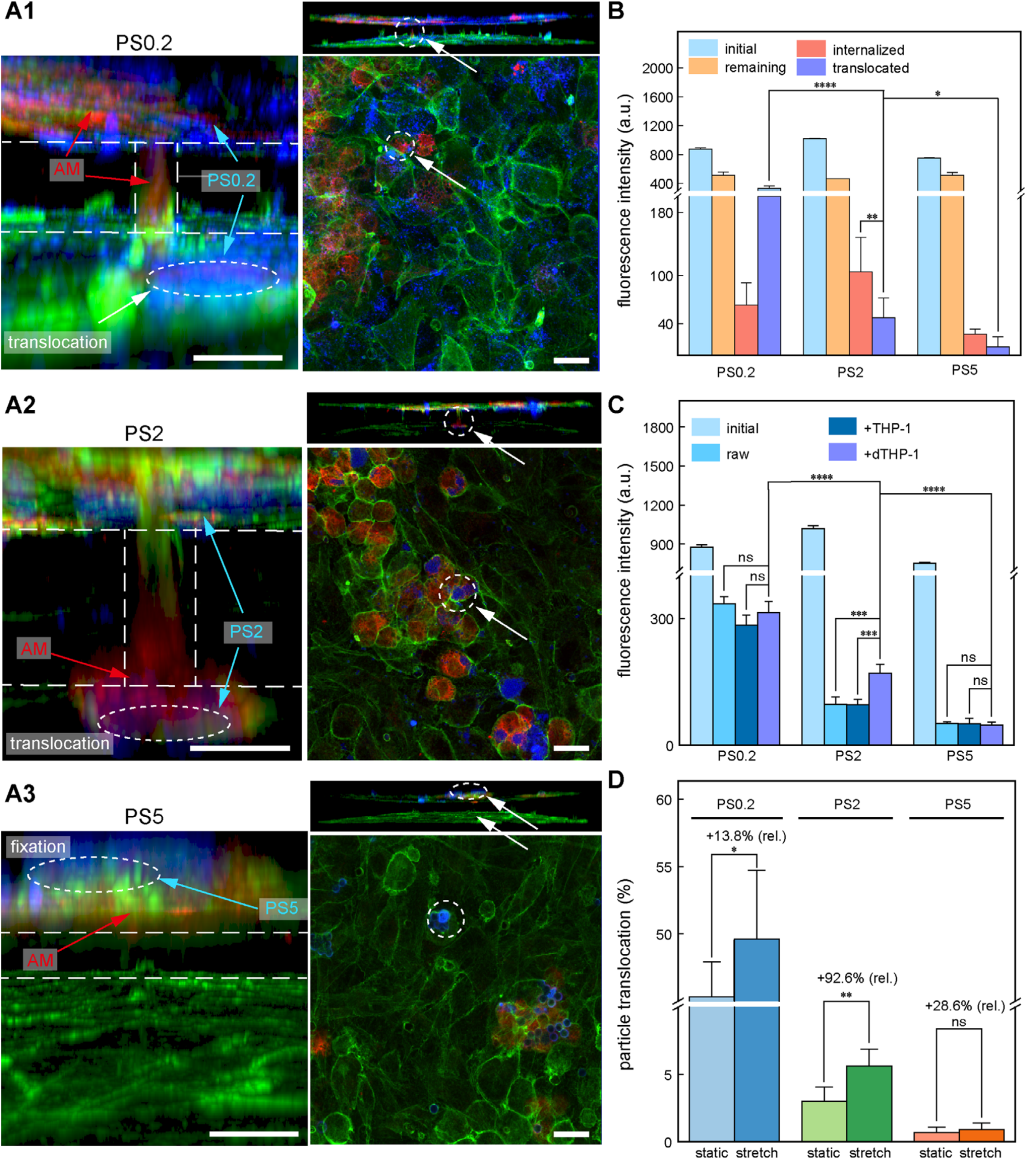
Size‐dependent translocation of PM_2_._5_ particles across the ABB is regulated by macrophages and cyclic strain. A1–A3) CLSM images showing the localization and translocation of PS0.2, PS2, and PS5 particles at the ABB following dTHP‐1 cells addition. Z‐projections and orthogonal views depict particle distribution relative to F‐actin (green), macrophages (red), and nuclei (blue). White arrows indicate translocated particles (scale bar, 10 µm). B) Quantitative fluorescence analysis of particle distribution under static, macrophage‐free conditions, showing initial apical content, residual apical particles (remaining), membrane‐retained fraction (internalized), and basal translocation (translocated) (*n* = 6 independent chambers; mean ± s.d.; ^***^ *p*< 0.001). C) Basal‐side fluorescence intensity indicating translocation efficiency of particles under three conditions: no macrophages (raw), undifferentiated THP‐1 cells (+THP‐1), and differentiated THP‐1 cells (+dTHP‐1) (*n* = 6 independent chambers; ^***^ *p* < 0.001, ^****^ *p*  < 0.0001). D) Translocation rates of PS0.2, PS2, and PS5 under static and 5% cyclic strain, showing relative enhancement under mechanical stimulation (*n* = 6 independent chambers; mean ± s.d. ^*^ *p* < 0.05, ^**^ *p* < 0.01).

To investigate the effect of particle size on macrophage‐mediated translocation across the ABB, we exposed an in vitro ABB model to polystyrene particles with diameters of 0.2, 2, and 5 micrometers, corresponding to ultrafine, fine, and coarse particulate matter, respectively, under 5 percent cyclic strain for 2 h. A549 and HUVEC cell boundaries were stained with phalloidin (green), dTHP‐1 cells with CytoTrace (red), and PS particles appeared in blue (**Figure** **4**A1–A3). Although variations in macrophage density may appear in these final images due to differences in subsequent cell adhesion and particle interaction, the initial seeding density of dTHP‐1 cells was consistent across all experimental conditions, as shown in Figure S9 (Supporting Information). PS2 particles aggregated along the epithelial surface and within intercellular spaces, showing clear colocalization with cell membranes of dTHP‐1 cells. Enlarged views revealed dTHP‐1 cells containing PS2 migrating across the porous membrane toward the endothelial layer, with PS2 signals observed on the basal side overlapping with macrophage membranes, indicating particle translocation facilitated by cell migration. In contrast, PS0.2 particles were broadly distributed throughout the ABB, including the basal side, but exhibited minimal colocalization with dTHP‐1 cells, indicating that their translocation was independent of macrophage phagocytosis. PS5 particles remained sparsely localized on the apical epithelial surface, showing neither basal signals nor interaction with dTHP‐1 cells. In the basal supernatant, dTHP‐1 cells containing PS0.2 or PS2 particles were detected, whereas no PS5‐associated cells were observed, supporting the notion that translocation of micron‐sized particles requires both appropriate size and macrophage involvement.

Under static conditions, PS0.2 particles exhibited the highest translocation rate of 38.25%, while PS2 and PS5 showed significantly lower values of 4.63% and 1.42%, respectively (Figure 4C; *n* = 6 independent chip chambers; mean ± s.d.). PS2 also showed greater epithelial retention of 12.35% compared to 3.49% for PS5, suggesting more pronounced interaction and partial internalization by epithelial cells. To assess macrophage involvement, particle translocation was compared in the presence of undifferentiated THP‐1 (+THP‐1) and differentiated dTHP‐1 cells. Only the dTHP‐1 group showed a marked increase in PS2 translocation, from 9.5% to 17.8%, while PS0.2 and PS5 remained unchanged across conditions, confirming that macrophage activation is required for efficient PS2 transport (Figure 4D). We further quantified the relative enhancement of translocation across different particle sizes under cyclic strain, both in the presence and absence of dTHP‐1 cells (Figure 4E). While all particles demonstrated increased translocation, the relative enhancement was most pronounced for PS2 (+92.6%), compared to PS0.2 (+13.8%) and PS5 (+28.6%). These findings indicate that particle translocation is jointly regulated by size and biological transport mechanisms, with PS2 achieving the most effective delivery when both phagocytosis and mechanical facilitation are present.

In summary, our study demonstrates the translocation capacities of PM_2_._5_ particles of different sizes across the in vitro ABB, providing valuable insights for both environmental health risk assessment and the development of macrophage‐assisted pulmonary drug delivery strategies.

## 3 Discussion

**Figure 5:**
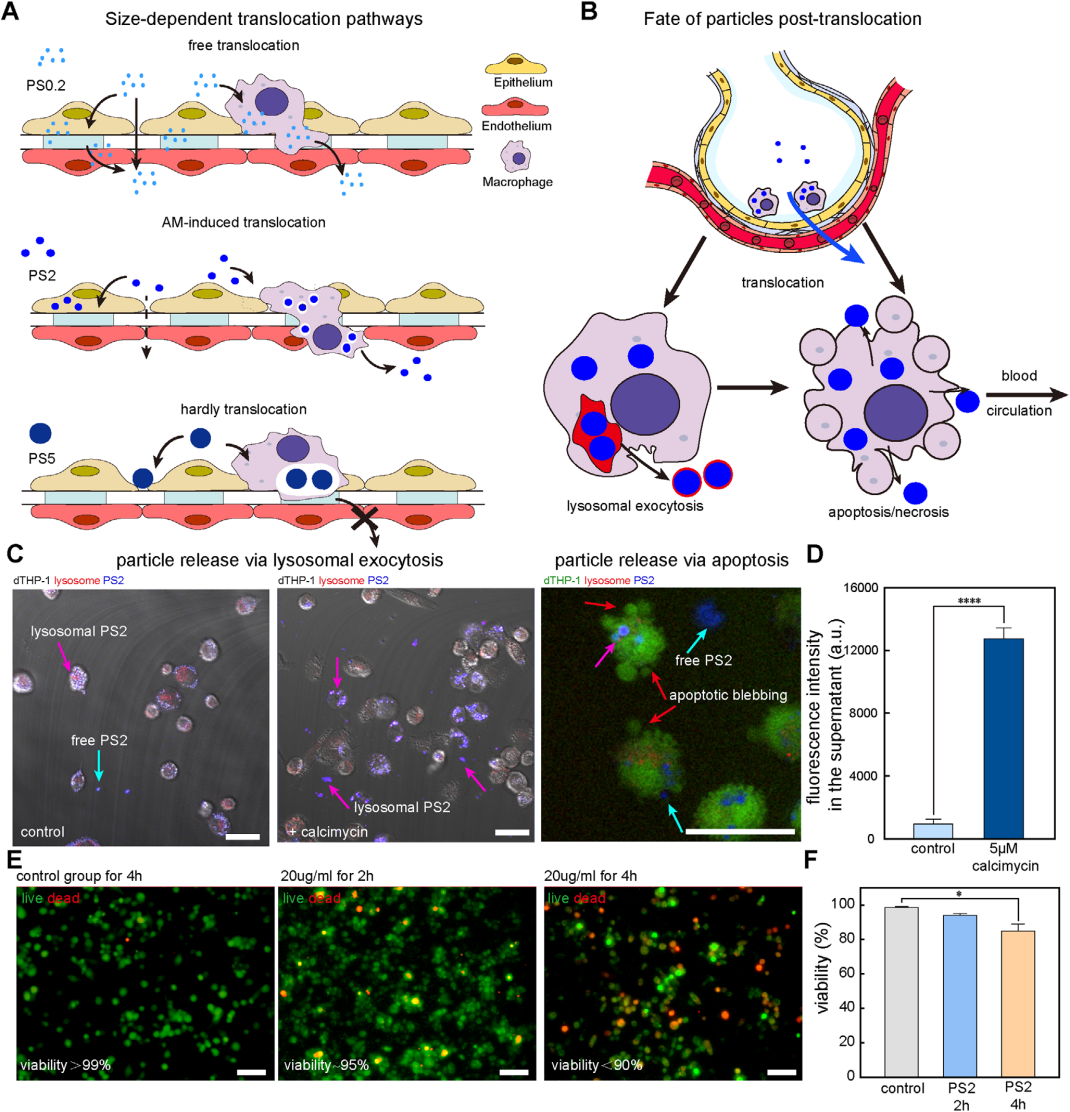
Proposed mechanisms of size‐dependent PM_2_._5_ translocation and the subsequent fate of phagocytosed particles. A) Schematic of size‐dependent translocation pathways across the air‐blood barrier. B) Schematic of the subsequent fate of particles after macrophage‐mediated translocation, showing two primary release pathways: active release via lysosomal exocytosis and passive release following apoptosis/necrosis. C) Live‐cell imaging demonstrating that stimulation with 5 µm A23187 induces both exocytosis and apoptosis in particle‐laden dTHP‐1 cells. Macrophages were pre‐stained with a green plasma membrane dye (CytoTrace). PS2 particles (blue) co‐localized with lysosomes (red) are visible inside cells (magenta arrows). Upon stimulation, independent particles are released into the extracellular space (cyan arrows), and cells undergo apoptosis, characterized by membrane blebbing (red arrows) (scale bar, 20 µm). D) Quantification of particle release by measuring the fluorescence intensity of the supernatant, showing a significant increase (*n* = 6 independent chambers, ^****^ *p* < 0.0001) after A23187 treatment. E) Representative images of a separate live/dead viability assay of macrophages following exposure to 20 µg mL^−1^ PS2 particles. In this assay, live cells are identified by cytoplasmic Calcein‐AM staining (green), while dead cells are stained with Propidium Iodide (red) (scale bar, 20 µm). F) Quantification of cell viability from the live/dead assay, showing a time‐dependent decrease upon particle exposure (*n* = 6 independent chambers, ^*^ *p* < 0.05).

To clarify the complex interplay among particle size, the mechanical environment, and cellular behavior, we propose a size‐dependent transport model for PM_2_._5_ translocation across the barrier (**Figure** **5**A). For submicron PS0.2 particles, their small size allows for passive transport through intercellular gaps widened by cyclic strain, a process independent of macrophage involvement. In contrast, large PS5 particles are unable to traverse the physical constraints of the epithelium layer, even after phagocytosis, thus exhibiting the lowest translocation efficiency. PS2 particles, however, are transported by a distinct mechanism in which they are efficiently phagocytosed by macrophages, and this active transport is synergistically enhanced by cyclic strain, resulting in the highest relative enhancement of translocation.

Furthermore, a central finding of this study is that translocated particles are subsequently released by macrophages into the subendothelial compartment. This secondary release critically shapes particle redistribution and biological activity in vivo.^[^ ^24^ ^]^ Further analyses indicate that the process is driven by at least two distinct mechanisms, including active lysosomal exocytosis and passive release upon macrophage death (Figure 5B). We first assessed lysosomal exocytosis as a potential active transport route, a process for releasing indigestible cargo that is well documented in nanoparticle studies.^[^ ^66^, ^67^ ^]^ To specifically induce lysosomal exocytosis in dTHP‐1 cells, we employed the calcium ionophore A23187.^[^ ^68^ ^]^ As shown in Figure 5C and Videos S3 and S4 (Supporting Information), A23187 treatment markedly increased the number of extracellular PS2 particles compared with untreated controls, with many particles visibly associated with lysosomal membranes (pink arrows). Higher concentrations of A23187 appeared to accelerate the transfer and exocytosis of intracellular particles toward the cell membrane. Quantification of particle fluorescence in the culture supernatant confirmed this observation, demonstrating a significant elevation of PS2 release upon A23187 stimulation (Figure 5D). These results establish that lysosomal exocytosis is an efficient route for macrophages to actively release internalized particles following their translocation across the ABB.

Then, we evaluated passive release via macrophage death, a process governed by the survival status of particle‐loaded macrophages. Using live/dead cell staining, we assessed the cytotoxicity of PS2 particles toward macrophages. As shown in (Figure 5E,F), exposure to 20 µg mL^−1^ PS2 particles for 4 h reduced cell viability to below 90% (*n* = 3, independent fields, P < 0.05), compared with >99% viability in DMSO‐treated controls. This finding indicates that PS2 particles exert intrinsic cytotoxicity, progressively inducing macrophage death. Consistent with this, fluorescence microscopy revealed apoptotic and necrotic cells displaying disrupted membrane integrity, leading to passive release of internalized PS2 particles into the surrounding environment (Figure 5C, right panel, blue arrows).

In summary, these results demonstrate that macrophage‐mediated particle release is a multifaceted process where active lysosomal exocytosis and passive, cytotoxicity‐induced discharge may operate in concert under different physiological or pathological conditions, determining the fate of microparticles in vivo. However, capturing these synergistic dynamics in organ‐scale microfluidic models remains challenging, and further investigation is needed to evaluate the long‐term risks of microparticle exposure.

Although significant research has focused on the adverse health effects of PM_2_._5_, a critical gap persists in understanding the specific mechanisms that govern their translocation across the ABB. From airway deposition to interaction with the ABB and eventual translocation, particles undergo multiple, dynamic biological interactions.^[^ ^25^, ^26^, ^69^ ^]^ These events occur rapidly and at microscopic scales, making in vivo observation difficult.^[^ ^38^, ^53^ ^]^ In our previous work, PS2 particles were detected in blood vessels and extrapulmonary organs of live mice using real‐time two‐photon microscopy, yet the mechanism of translocation has remained unclear.

Inspired by the primary role of alveolar macrophages in clearing inhaled particulates, we propose that the translocation of micro‐sized particles across the ABB is predominantly mediated by macrophages. Previous studies have demonstrated that alveolar macrophages can detect microparticles or liposomes ranging from 1 to 5 µm, capturing and phagocytosing them more effectively than nanoparticles.^[^ ^45^, ^70^ ^]^ Additionally, the uptake behavior of alveolar macrophages toward PS particles of different diameters (0.2, 0.5, 1.0, 6.0, and 10.0 µm) showed that 1 µm microspheres exhibited the highest uptake, overlapping with the upper size range of PM_2_._5_ particles.^[^ ^71^ ^]^ We hypothesize that the preferential recognition of particles within this size range by AMs plays a critical role in their translocation^[^ ^72^ ^]^

Therefore, PMA‐induced THP‐1 cells were employed as an AM model and incorporated into the co‐culture system to simulate macrophage‐mediated transport mechanisms.^[^ ^60^ ^]^ Notably, our quantitative analysis revealed that dTHP‐1 cells mediate the translocation of large PM_2_._5_ particles across the ABB under cyclic mechanical strain, primarily through a phagocytosis‐dependent mechanism. When applied separately, cyclic strain increased particle translocation from a static baseline of 1.5–5.2%, whereas the presence of macrophages resulted in a rate of 3.9%. Under combined conditions, the translocation rate rose to 15.5%, indicating a strong synergistic interaction between biomechanical stress and macrophage‐mediated transport rather than a simple additive effect. Our model represents a substantial advance over existing platforms by functionally integrating elements that were previously examined separately, including out‐of‐plane dynamic mechanical strain and immune cell involvement, thereby achieving greater physiological relevance and system complexity in the study of PM translocation.

Moreover, the strong synergistic effect observed in our study implies that cyclic stretching functions not just as a physical force, but also as a key biological signal that directly modulates macrophage function to enhance particle translocation. During respiration, alveolar macrophages are exposed to a dynamic mechanical microenvironment characterized by rhythmic stretching. There is growing evidence that macrophages can directly sense and respond to these mechanical stresses via mechanosensors on their cell surface, such as integrins and piezo‐type ion channels (e.g., Piezo1).^[^ ^73^ ^]^ This mechanotransduction signaling profoundly influences macrophage function, with physiological cyclic stretching shown to enhance phagocytic capacity through the formation of phagocytic cups and actin remodeling.^[^ ^55^ ^]^ Meanwhile, mechanical stress can also serve as a chemotactic‐like signal, activating the migratory program of macrophages and enabling more effective patrolling and movement within tissues, while also regulating macrophage polarization phenotypes.^[^ ^74^ ^]^ Consequently, the marked enhancement of macrophage‐mediated particle translocation observed under cyclic strain likely results from two concurrent processes, in which stretching compromises the epithelial barrier to facilitate cell migration, while simultaneously activating macrophages to enhance their capacity for phagocytosis and barrier traversal. Indeed, the enhancement of migratory activity under strain was validated, as shown in Figure S6 (Supporting Information). This synergy between mechanical forces and immune cell behavior reveals that respiratory stretching itself is a key regulator in the in vivo clearance and translocation of particulate matter.

A critical aspect of this study is the physiological relevance of the in vitro ABB model. To quantitatively benchmark our system, we compared its barrier properties with established in vitro models and physiological standards (Table S1, Supporting Information). Although the TEER of our tri‐culture model reached ≈108 Ω·cm^2^, which is lower than that of human lung tissue or models established with primary alveolar cells, it is consistent with values reported for other dynamic models based on the A549 cell line and possesses a molecular permeability comparable to other models, demonstrating the effectiveness of the membrane barrier. This indicates that our platform establishes a functional and reproducible barrier with sufficient integrity to prevent the passive leakage of micron‐sized particles, thereby providing a valid baseline for studying active, cell‐mediated transport. For the immune component, we employed PMA‐differentiated THP‐1 macrophages as a surrogate, a model that has been widely used in lung barrier tri‐culture systems to study inflammatory responses, particle phagocytosis, and trans‐barrier processes.^[^ ^50^ ^]^

Furthermore, accurate quantification of the particle load is essential for enhancing the reliability and physiological relevance of our investigation into the translocation mechanism. To prevent differences in the initial phagocytosis rate from confounding the translocation results, particle uptake was quantified under near‐saturated conditions. A 2–4 h incubation allowed internalization to reach a plateau, as evidenced by the comparable total internalized volume shown in Figure S3A (Supporting Information). This design ensured a uniform particle load across macrophages, enabling the effects of particle size on translocation to be evaluated independently of uptake kinetics. In the exposure study, a particle concentration of 20 µg mL^−1^ corresponded to a surface dose of ≈2.9 µg cm^−^^2^ at the ABB. This level is physiologically relevant, as it falls within the range of lung surface deposition observed in animal models following acute, high‐dose aerosol exposure (typically 5–10 µg cm^−^^2^).^[^ ^75^ ^]^ In vitro studies have also employed doses up to ≈21 µg cm^−^^2^ of diesel exhaust particles to examine the translocation process,^[^ ^76^ ^]^ and even higher concentrations (100–200 µg mL^−1^) to evaluate acute inflammatory responses.^[^ ^77^ ^]^ Therefore, the selected dose represents a balanced condition that enables mechanistic investigation of translocation while minimizing the non‐specific cytotoxicity associated with higher exposures. Notably, it is consistent with the dosage used in our previous foundational work.^[^ ^37^ ^]^

Nevertheless, we must also acknowledge the inherent limitations of our model. A549 cells mainly exhibit an ATII‐like phenotype. They can form functional monolayers, express junctional proteins (e.g., ZO‐1), and perform key functions such as particle uptake and inflammatory signaling, but cannot fully reproduce the large surface area of ATI cells or the native surfactant layer.^[^ ^78^ ^]^ Likewise, PMA‐differentiated THP‐1 macrophages, derived from a leukemia cell line, differ from primary alveolar macrophages in phenotype, phagocytic ability, and cytokine secretion (e.g., IL‐1β and TNF‐α) upon particle exposure.^[^ ^79^ ^]^ While the model may not fully reproduce the complex biological responses of primary cells during particle translocation, the use of dTHP‐1 macrophages is justified given the difficulty of obtaining primary alveolar macrophages. To date, dTHP‐1 cells have been widely recognized as suitable surrogates,^[^ ^80^ ^]^ combining the essential macrophage functions with the stability and reproducibility needed to clearly distinguish the effects of mechanical strain and macrophage activity. Overall, this integrated model achieves a balance between physiological relevance and experimental control, providing a robust platform for investigating the biomechanical‐immune coupling that governs micron‐sized particle translocation. Looking ahead, incorporating primary or stem‐cell‐derived alveolar cells could further enhance the physiological fidelity of the model, offering a more authentic representation of the alveolar barrier and enabling patient‐specific studies on the interplay between genetic predisposition and particle‐induced pathology, as recently established in the field.^[^ ^81^, ^82^ ^]^

The findings of this study provide a mechanistic framework with significant implications for both environmental toxicology and advanced therapeutic design. On one hand, the discovery of a highly efficient, mechanically regulated “Trojan horse” translocation pathway for MPs provides a plausible cellular basis for the established epidemiological links between particle exposure and extrapulmonary diseases, such as atherosclerosis, neuro‐inflammation, and systemic autoimmune responses.^[^ ^83^ ^]^ Furthermore, the role of macrophages as carriers transporting particles to distal organs may exacerbate systemic toxicological effects, suggesting that the translocation potential of particles, in addition to their mass concentration, is a critical determinant of health risk. On the other hand, this same mechanism can be harnessed for advanced therapeutics, positioning alveolar macrophages as ideal drug delivery systems.^[^ ^84^ ^]^ This strategy is particularly promising for pulmonary and systemic diseases. For instance, compared to nanoparticles, micron‐sized drug formulations offer a higher loading capacity, are more readily deposited on the alveolar surface for localized action, and can minimize the risk of premature systemic metabolism. By leveraging macrophage‐mediated transport, therapeutic agents for diseases such as cystic fibrosis, acute respiratory distress syndrome (ARDS), and even lung cancer could be delivered more effectively to deep lung tissues or be transported into the circulation for systemic action.^[^ ^85^, ^86^ ^]^ Our in vitro platform provides a powerful and physiologically relevant tool for the screening and optimization of such next‐generation, macrophage‐targeted inhalation therapies.

## 4 Conclusion

In this work, we reveal the cellular mechanisms underlying the translocation of larger PM_2_._5_ particles across the ABB. By integrating a triple co‐culture model consisting of epithelial cells, endothelial cells, and phagocytic macrophages, with cyclic mechanical strain mimicking respiratory motion, we established an in vitro ABB platform with robust barrier functionality. Using this platform, we demonstrated that translocation is strongly size‐dependent. Ultrafine particles (PS0.2) cross the barrier primarily through passive diffusion, intermediate‐sized particles (PS2) are efficiently transported via macrophage‐mediated translocation enhanced by mechanical strain, a process confirmed through pharmacological inhibition to be driven by phagocytosis, and larger particles (PS5) show minimal transport due to their size limitations. Furthermore, our investigation into the subsequent fate of translocated particles suggests they are released from macrophages via both active lysosomal exocytosis and passive, cell death‐mediated pathways. Our findings reveal a key role between mechanical forces and immune cell activity in facilitating the translocation of fine particulate matter, bridging the gap in the mechanistic understanding of PM_2_._5_ transport by elucidating a more complete translocation pathway from initial uptake to subsequent release. Ultimately, this work not only provides a crucial toxicological insight into how inhaled microparticles contribute to systemic disease but also paves the way for developing novel therapeutic strategies that leverage this natural biological pathway for targeted drug delivery.

## 5 Experimental Section

### Fabrication of the In Vitro Lung‐on‐a‐Chip

The lung chip array consists of a fluidic module, a pneumatic driving module, and a flexible PDMS (polydimethylsiloxane) membrane sandwiched between them (Figure 1B). The fluidic module was composed of two PDMS layers with a porous, flexible membrane embedded in between and bonded together. The upper PDMS layer contains a central inlet port (3 mm in diameter) for the introduction of culture medium or microparticles. The lower layer includes a medium reservoir aligned with the inlet to support subsequent cell culture. The pneumatic module comprises a vacuum channel and a 30–40 µm thick PDMS membrane, which was irreversibly bonded to the driving layer to ensure structural integrity. Both fluidic and pneumatic components were fabricated using soft lithography. Briefly, PDMS base and curing agent (Sylgard 184, Dow Corning, USA) were mixed at a 10:1 weight ratio, degassed, and poured into pre‐patterned silicon molds. These molds were prepared via maskless soft lithography: SU‐2075 (MicroChem, USA) photoresist was spin‐coated onto silicon wafers at 1400 rpm, followed by a 15‐min soft bake and a 45‐min post‐bake. UV exposure was performed using a maskless exposure system (SF‐100, Intelligent Micro Patterning, USA), after which the uncured photoresist was removed by development. Finally, the PDMS was cured in the molds at 65 °C for 12 h to form the desired microstructures.

The porous PDMS membrane that supports cell growth was fabricated using silicon etching and spin‐coating. A 4‐inch silicon wafer was patterned with micropillars (8 µm diameter, 20 µm height) via deep reactive ion etching (DRIE) using CF_4_ and SF_6_ gases. To enable easy demolding, the etched surface was made hydrophobic through immersion in diluted octadecyltrichlorosilane (OTS, Aladdin, China) for 40 min, followed by rinsing with hexadecane, acetone, and ethanol (all from Aladdin, China), and then dried. For membrane formation, ≈4 mL of PDMS was deposited onto the mold and spin‐coated at 2500 rpm for 5 min, forming a thin film over the pillar array. After curing and demolding, this process yields a membrane with through‐holes. Then the membrane and bottom plate were treated with oxygen plasma (VP‐R3, Shanzhun Technology, China) to achieve irreversible bonding and simultaneous release from the mold. The top plate was then reversibly attached to complete the lung chip array.

### Cell Culture and Co‐Culture Experiments

To model the co‐culture system, A549 lung epithelial cells (ATCC, Manassas, VA, USA) and human umbilical vein endothelial cells (HUVECs, Procell, China) were used to represent the epithelial and endothelial layers, respectively. A549 cells were cultured in F‐12K medium supplemented with 10% fetal bovine serum (FBS) and 1% penicillin‐streptomycin (all reagents from Procell, China). HUVECs were cultured in Endothelial Cell Medium (ECM) consisting of basal medium supplemented with 5% FBS, 1% penicillin–streptomycin, and 1% ECGS (all from ScienCell, USA). Cells were incubated at 37 °C with 5% CO_2_ under sterile conditions. Both cell types were passaged 20–30 times prior to seeding into the microfluidic device to ensure stable proliferation and viability.

To induce macrophage‐like polarization, THP‐1 cells (Procell, China) were seeded at a density of 5  ×  10⁵ cells per T25 flask in RPMI 1640 medium supplemented with 10% FBS, 1% penicillin–streptomycin (Procell, China), and 100 ng mL^−1^ phorbol 12‐myristate 13‐acetate (PMA, Tocris, UK). After 48 h of stimulation, cells exhibited adherent morphology characteristic of PMA‐polarized THP‐1 cells. Cells were then washed twice with PBS, detached using trypsin‐EDTA (Procell, China) for 5 min, and resuspended in fresh medium. These polarized cells were either introduced into the apical channel of the lung chip or seeded onto standard culture dishes for particle uptake assays. Epithelial and endothelial cells were seeded into their respective microchannels at 2 × 10⁶ cells mL^−1^. Channels were pre‐coated with 50 µg mL^−1^ human fibronectin (Procell, China) to enhance cell adhesion. Medium was replaced every 12 h initially, and every 6 h once both monolayers reached confluence.

### Stretching Protocol

Cyclic mechanical strain was applied using a programmable pressure controller (OB1+ MK3, Elveflow, France), which allowed precise regulation of strain amplitude and waveform to simulate both physiological (5–12%) and pathological breathing patterns. To characterize the strain–pressure relationship, porous PDMS membranes without cells were first filled with culture medium and subjected to varying levels of negative pressure. Strain was quantified by measuring the center‐to‐center distance between the first and fifth pores along a straight row using ImageJ software, allowing for enhanced measurement sensitivity. This measurement was repeated at six locations for each pressure level, and the average value was used to represent the strain at that condition. After calibration, epithelial and endothelial cells were seeded on opposite sides of the membrane. Once confluent monolayers formed, the culture medium on the apical side was replaced to completely cover the cell monolayer, and 50 µL of medium was added to the basal side to form a suspended droplet. The fluidic and pneumatic layers were then carefully aligned and reversibly bonded. Membrane deformability and structural integrity after cell seeding were confirmed under cyclic strain using fluorescence microscopy (IX83, Leica, Germany) and confocal imaging (LSM780, Leica, Germany).

### Immunofluorescence

To assess cell–cell junction integrity and phenotype, immunofluorescence staining was performed on epithelial and endothelial monolayers. Cells were first washed with PBS and fixed in 4% paraformaldehyde (Sigma–Aldrich, USA) for 12 min at room temperature. After rinsing with PBS, permeabilization was conducted using 0.2% Triton X‐100 (Sigma–Aldrich, USA) for 10 min. To block nonspecific binding, samples were incubated for 30 min in PBS containing 5% fetal bovine serum (FBS) and 1% bovine serum albumin (Aladdin, China). Primary antibodies against E‐cadherin (ab40772, Abcam, UK; 1:200) and VE‐cadherin (ab232880, Abcam, USA; 1:100) were diluted in blocking buffer and applied for 2 h at room temperature. After washing, secondary antibodies (Goat anti‐rabbit Alexa Fluor 594 and Alexa Fluor 488, Invitrogen, USA; 1:200) were added and incubated for 1 h at 20 °C in the dark. Samples were then washed three times with blocking buffer and mounted with DAPI‐containing medium (P0131, Beyotime, China). Images were acquired using a confocal laser scanning microscope (LSM 780, Zeiss, Germany).

### TEER Measurement

The transepithelial electrical resistance (TEER) of the cell cultures grown on the membrane was measured using the Millicell ERS system (Millicell ERS‐2, Millipore, USA). TEER was calculated by multiplying the electrical resistance by the effective surface area of the membrane (cm^2^). The TEER value of the blank PDMS membrane was determined to be 20±7.5 (Ω·cm^2^), which was then subtracted from the TEER value of the cell‐covered membrane.

### Permeability Assay

12 h after the formation of confluent bilayers, the integrity of the barrier was assessed by measuring the transport of two hydrophilic tracers of different molecular weights. To initiate the assay, the basal chamber was filled with fresh culture medium (A549 and HUVEC medium, 1:1, v/v). A solution containing either 10 µg mL^−1^ sodium fluorescein (NaFlu, 376 Da) or 1 mg mL^−1^ FITC‐dextran (4 kDa, both from Maokang Biotechnology, Shanghai, China) was added to the apical chamber. The chip was incubated at 37 °C and 5% CO2 under static conditions. After 2 h, 20 µL of medium was collected from both the apical and the basal chamber. Following a 1:10 (v/v) dilution, the fluorescence intensities of the collected samples were measured using a microplate reader with excitation/emission wavelengths of 460/515 nm for both FITC‐tagged tracers. The concentration of the tracers in the samples was determined from a standard curve. Finally, the apparent permeability coefficient (*P*_app_) in cm/s was calculated using the following equation:

(1) $$P_{\mathrm{app}}=\frac{V_{A}\times c_{A}}{A\times T\times c_{0}}$$

where *V*_A_ is the volume of the acceptor chamber (20 µL), *C*_A_ is the final concentration of the tracer in the basal chamber (µg/mL or mg/mL), *A* is the surface area of the membrane (0.07 cm^2^), *T* is the total incubation time (7200s), and *C*_0_ is the initial concentration in the apical chamber (µg/mL).

### Particle Translocation Experiments

dTHP‐1 cells were introduced into the apical chamber either directly or following centrifugation and detachment, after monolayer confluence was achieved on both sides of the membrane. The device was then incubated for an additional 12 h under standard conditions (37 °C, 5% CO_2_) to evaluate particle translocation in the presence or absence of dTHP‐1 cells. For particle tracking, laser scanning confocal microscopy (CLSM; Zeiss LSM 780) was used. Five representative regions with intact and confluent cell layers were selected for imaging. Fluorescence channels at 405, 488, and 594 nm were used to visualize particles, A549 cells, and either HUVECs or dTHP‐1 cells, respectively. Prior to fixation, samples were washed with PBS to remove free or loosely bound particles. For static particle uptake analysis, cells were fixed in 4% paraformaldehyde; for dynamic tracking of dTHP‐1 behavior, fixation was omitted to preserve viability. Z‐stack images were acquired using a 20× objective with an optical section thickness ≤1 µm. Cumulative fluorescence intensity across the z‐stacks was quantified to assess intracellular particle uptake. In parallel, the fluorescence intensity of the basal‐side medium—reflecting translocated particles—was measured using a microplate reader (Safire2, Tecan) at 405 nm excitation and 450 nm emission. Translocation efficiency was calculated as the percentage of fluorescence in the basal chamber relative to that initially applied to the apical side. At least three replicates were analyzed per condition.

### Cellular Dynamics Tracking

To investigate how dTHP‐1 cells facilitate PM_2_ translocation, the real‐time behavior of individual dTHP‐1 cells was monitored using laser scanning confocal microscopy (CLSM; Zeiss LSM 780). For visualization, dTHP‐1 cells were labeled with a red fluorescent dye, the A549/HUVEC co‐culture was stained green, and blue fluorescent PS particles were used. Time‐lapse Z‐stack images were acquired at regular intervals. The chip was maintained in a temperature‐ and CO_2_‐controlled chamber integrated with the microscope to ensure cell viability during imaging. The resulting image sequences were processed to generate videos using ZEN software (Zeiss, Germany).

### Particle Exocytosis Assay

To validate the particle release pathway via lysosomal exocytosis, dTHP‐1 cells were first cultured in glass‐bottom confocal dishes and allowed to phagocytose 2 µm PS2 particles for 4 h. The cells were subsequently stained with a lysosomal probe (LysoTracker Red) and a plasma membrane dye (CytoTrace Green). For live‐cell imaging, the culture medium was replaced with pre‐warmed, phenol red‐free imaging medium supplemented with serum. The dish was mounted on a laser scanning confocal microscope (CLSM; Zeiss LSM 780) equipped with a chamber providing temperature (37 °C) and CO_2_ (5%) control. Time‐lapse Z‐stack images were acquired for 5 min to establish a baseline. Subsequently, the calcium ionophore Calcimycin (A23187) was added to a final concentration of 5 or 10 µm without interrupting the imaging, and the cellular response was monitored for an additional 20 min. A parallel control experiment was performed using an equivalent volume of the vehicle (DMSO). For quantification, the supernatant from separate wells was collected after treatment, and the fluorescence intensity of the released PS2 particles was measured using a microplate reader (Tecan M1000 Infinite) at an excitation/emission wavelength of 405/450 nm.

### Cell Viability Assay

To assess the cytotoxicity of the particles, dTHP‐1 cells were seeded in culture plates and incubated with 20 µg mL^−1^ of 2 µm PS2 particles for 2 and 4 h. A control group was incubated without particles for 4 h. After the incubation period, cell viability was determined using a Live/Dead Viability/Cytotoxicity Kit. Cells were washed once with PBS and then incubated with a solution containing Calcein‐AM (to stain the cytoplasm of live cells green) and Propidium Iodide (to stain the nuclei of dead cells with compromised membranes red). After a 15 min incubation at room temperature protected from light, the cells were imaged using a fluorescence microscope. Cell viability was quantified by counting the number of live (green) and dead (red) cells from at least three independent fields of view for each condition. The viability was expressed as the percentage of live cells relative to the total number of cells.

### Statistics

All data were presented as means ± standard deviation (SD). Two‐tailed unpaired Student's *t*‐test was used to assess the significance of differences. Statistical significance was defined as follows: ^*^ *p* < 0.05, ^**^ *p* < 0.01, ^***^ *p* < 0.001, ^****^ *p* < 0.0001. The replicate number “n” represents either an independent microfluidic chamber or an independent field of view, as specified in each figure legend.

## Conflict of Interest

The authors declare no conflict of interest.

## Supporting information



Supporting Information

Supplemental Video 1

Supplemental Video 2

Supplemental Video 3

Supplemental Video 4

## Data Availability

All relevant data are available in the manuscript or Supporting Information. Further inquiries can be directed to the corresponding author.
