# Supplementary material for: Cyclic Strain and Macrophage‐Mediated Transport Govern Micron‐Sized PM2.5 Translocation across the Air–Blood Barrier
Source: Adv Sci (Weinh). 2025 Oct 29;13(3):e11955. doi: 10.1002/advs.202511955 (PMC12806473; doi:10.1002/advs.202511955)
Supplement: Supplementary file 1 — Supporting Information [file ADVS-13-e11955-s005.docx]

Supporting Information

Cyclic strain and macrophage-mediated transport govern micron‑sized PM₂.₅ translocation across the air–blood barrier

Yongjian Li^1,2^*†, Jinlong Xu^1,2^†, Zujie Gao^1,2^ and Haosheng Chen^1,2^*

†YJ.L. and JL.X. contributed equally to this paper.

††Yongjian Li and Haosheng Chen are both corresponding authors.

^1^Department of Mechanical Engineering, Tsinghua University, Beijing 100084,P.R. China.

^2^State Key Laboratory of Tribology in Advanced Equipment, Tsinghua University, Beijing 100084,P.R. China.

***Correspondence:**

Yongjian Li − Department of Mechanical Engineering, Tsinghua University, Beijing 100084, P. R. China; orcid.org/0000-0002-3485-4253; E-mail: liyongjian@tsinghua.edu.cn;

Haosheng Chen − Department of Mechanical Engineering, Tsinghua University, Beijing 100084, P. R. China; orcid.org/0000-0002-1134-0989; E-mail: chenhs@tsinghua.edu.cn.


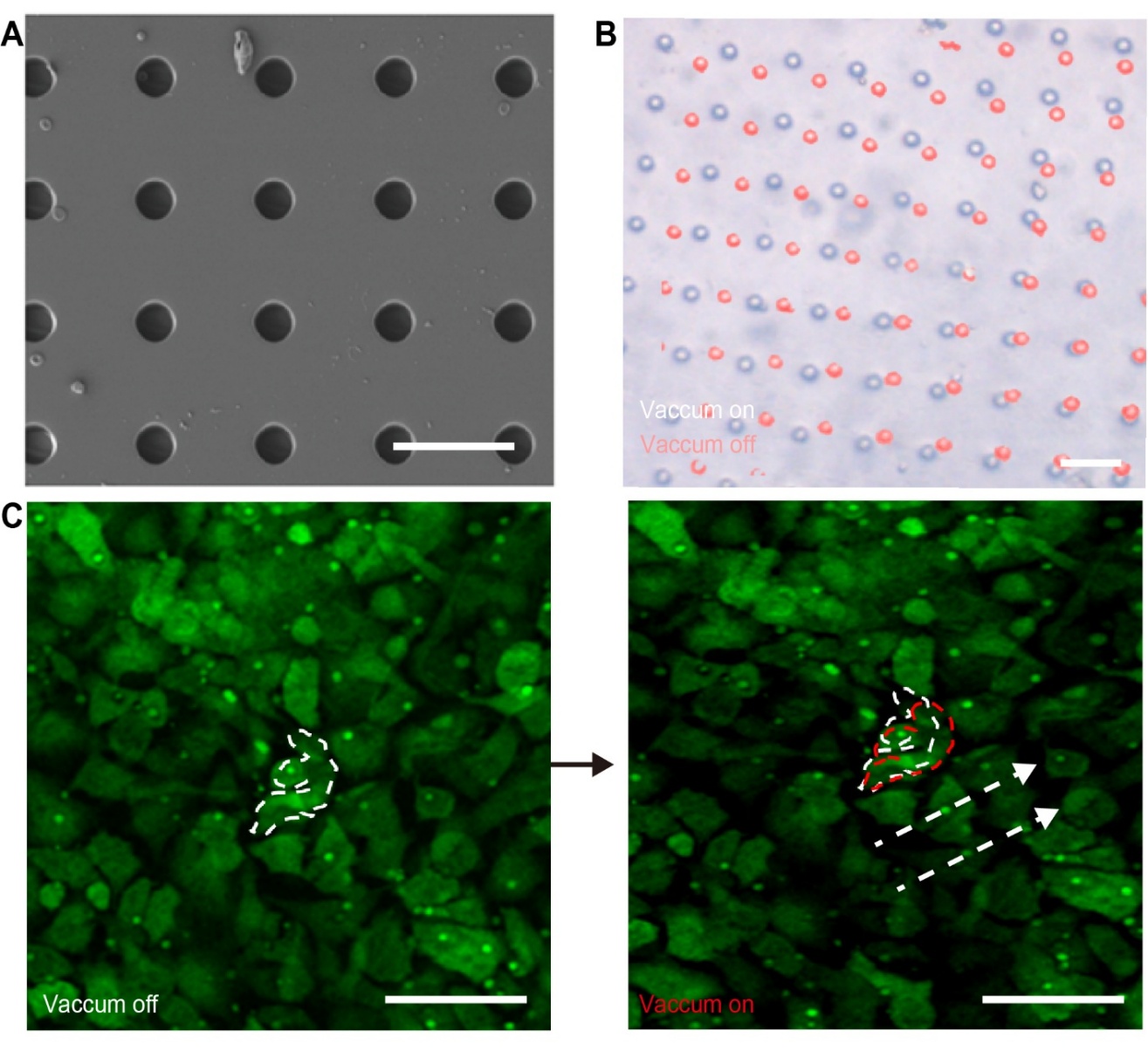


**Supplementary Figure S1. Structural and functional characterization of the ABB membrane.**(A) Scanning electron microscopy (SEM) image of the porous PDMS membrane used in the ABB device. The membrane contains uniformly spaced through-holes (12 μm in diameter), enabling direct interaction and particle exchange between epithelial and endothelial layers. (B) Overlay of optical images showing membrane pore alignment before (red) and after (blue) vacuum actuation, demonstrating lateral deformation under cyclic strain (scale bar, 50 μm). (C) Live-cell fluorescence images of A549 epithelial cells stained for F-actin (green), under static (Vacuum off) and strained (Vacuum on) conditions. Dotted outlines and arrows highlight stretching of cell boundaries and gap formation in response to cyclic mechanical loading (scale bar, 50 μm).

| **Cell type** | **Culture conditions** | **TEER（Ω·cm², time）** | **Small molecule permeability(NaFlu, % or *10^-7 cm/s)** | **Large molecule permeability(% or *10^-7 cm/s** | **Reference** |
| --- | --- | --- | --- | --- | --- |
| HATII+HPMEC | Transwell,  static | 1250 ± 350 (monoculture, 6days), ~2000 (coculture, 6dys） | N/A | N/A | Hermanns et al.Hermanns, et al. ^[1]^ |
| hAEpC | Transwell,  static | 2180±62 (18 days) | N/A | FD-4: 0.17±0.025，FD-40: 0.03±0.003 | Elbert et al.^[2]^ |
| 549/NCI-H441+HPMEC | LoC,  dynamic | ~800 (18 days) | N/A | BSA: 6%/hr | Huh et al.^[3]^ |
| hAEpC+HPMEC | LoC,  dynamic | 545.5 ± 2.86 (7days) | ~6.5±11 (static),  ~45±13 (dynamic) | 2.4±4.3 (static)；16±5.4 (stretch) | Stucki et al.^[4]^ |
| NCI-H441 | Transwell,  static | ~250 (12 days) | 3.5±0.65 | 0.48±0.087 | Suresh^[5]^ |
| NCI-H441，HPMEC-ST1.6R，dTHP-1 | LoC,  static | ~33.0, (5days) | 120 | N/A | Costa et al.^[6]^ |
| A549 | LoC,  static | 177.2±10.82 (21days) | ~2.5 | N/A | Nalayanda et al.^[7]^ |
| A549+pHUVEC | LoC,  dynamic | ~200 (dynamic, 6 days),  ~100 (static, 6 days) | 5.5% (static),  4.2% (dynamic） | N/A | Fu et al.^[8]^ |
| A549+pHUVEC | LoC,  static | 44.5±5 (6 days) | 220 | N/A | Sevinc et al.^[9]^ |
| A549+EA.hy 926+dTHP-1+HMC-1 | Transwell,  static | ~170，(7 days) | (fourculture model shows higher permeability) | N/A | Klein et al.^[10]^ |
| A549+pHUVEC+dTHP-1 | LoC,  dynamic | 108.0±15.3 (dynamic, 3 days) | 8.02±2.08 | 0.79±0.54 | **※this work** |

**Supplementary Table S1. Comparison of transepithelial electrical resistance (TEER) and apparent permeability (Papp) of small and large molecules across various in vitro lung models, detailing cell types and culture conditions.**

1. Values are extracted from peer-reviewed sources listed in the “Reference” column. Data are reported as mean ± SD (or mean alone) at the time point indicated.
2. TEER is expressed in Ω·cm² and was blank-corrected (cell-free insert/chip) and normalized to the effective membrane area. Where both static and dynamic conditions were reported, values are shown as “static” and “dynamic” . For epithelial–endothelial co-cultures, TEER represents the combined resistance across both layers.
3. Permeability is given as the apparent permeability coefficient (Papp, cm/s), calculated as Papp=(dQ/dt)/(A⋅C0). To aid comparison, values are reported in units of ×10⁻⁷ cm/s. “FD-X” denotes fluorescein-dextran of X kDa; mannitol is a small-molecule paracellular tracer.
4. Culture/readout abbreviations: **ALI** = air–liquid interface; **Transwell** = static porous support; **LoC/LOC** = microfluidic lung-on-chip; **dynamic** = cyclic mechanical strain.
5. Cell abbreviations: **HPAEpiCs/HAEC** = primary human alveolar epithelial cells; **HATII** = primary human alveolar type II cells; **A549** and **NCI-H441** = human alveolar/club epithelial lines; **HPMEC** or **HPMEC-ST1.6R** = human pulmonary microvascular endothelial (primary/line); **HUVEC/pHUVEC** = (primary) human umbilical vein endothelial cells; **dTHP-1** = differentiated THP-1 macrophage-like cells.
6. **n/a** or **n.d.** = not available/not determined.


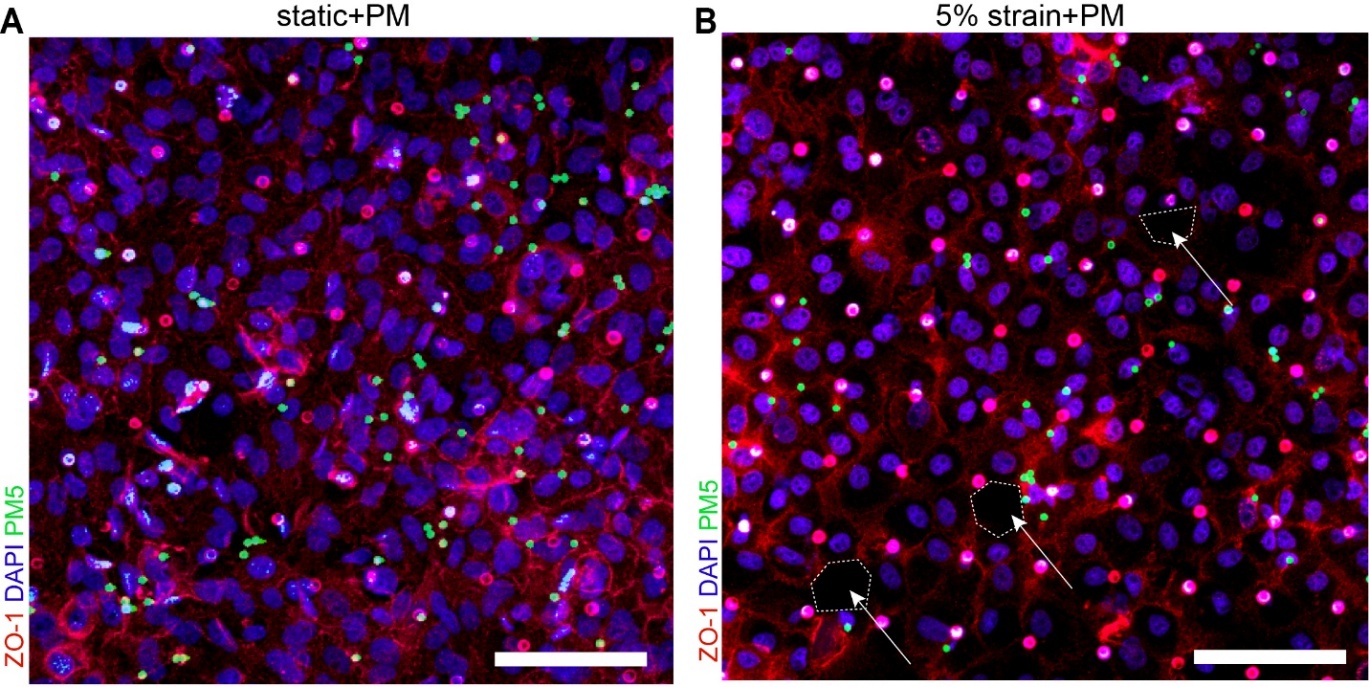


**Supplementary Figure S2**. Junctional disruption in A549 monolayers under PM exposure. (A) Confocal fluorescence image of A549 epithelial monolayer exposed to PM under static conditions. ZO-1 (tight junction marker) is stained in red, nuclei with DAPI in blue, and PM5 particles in green. The monolayer exhibits intact cell–cell junctions and uniform ZO-1 expression. (B) Under 5% cyclic strain, the same PM exposure led to visible disruption of ZO-1 distribution, with decreased signal intensity and widening of intercellular gaps (indicated by dashed outlines and arrows), suggesting that mechanical strain aggravates junctional impairment (scale bars, 50 μm).


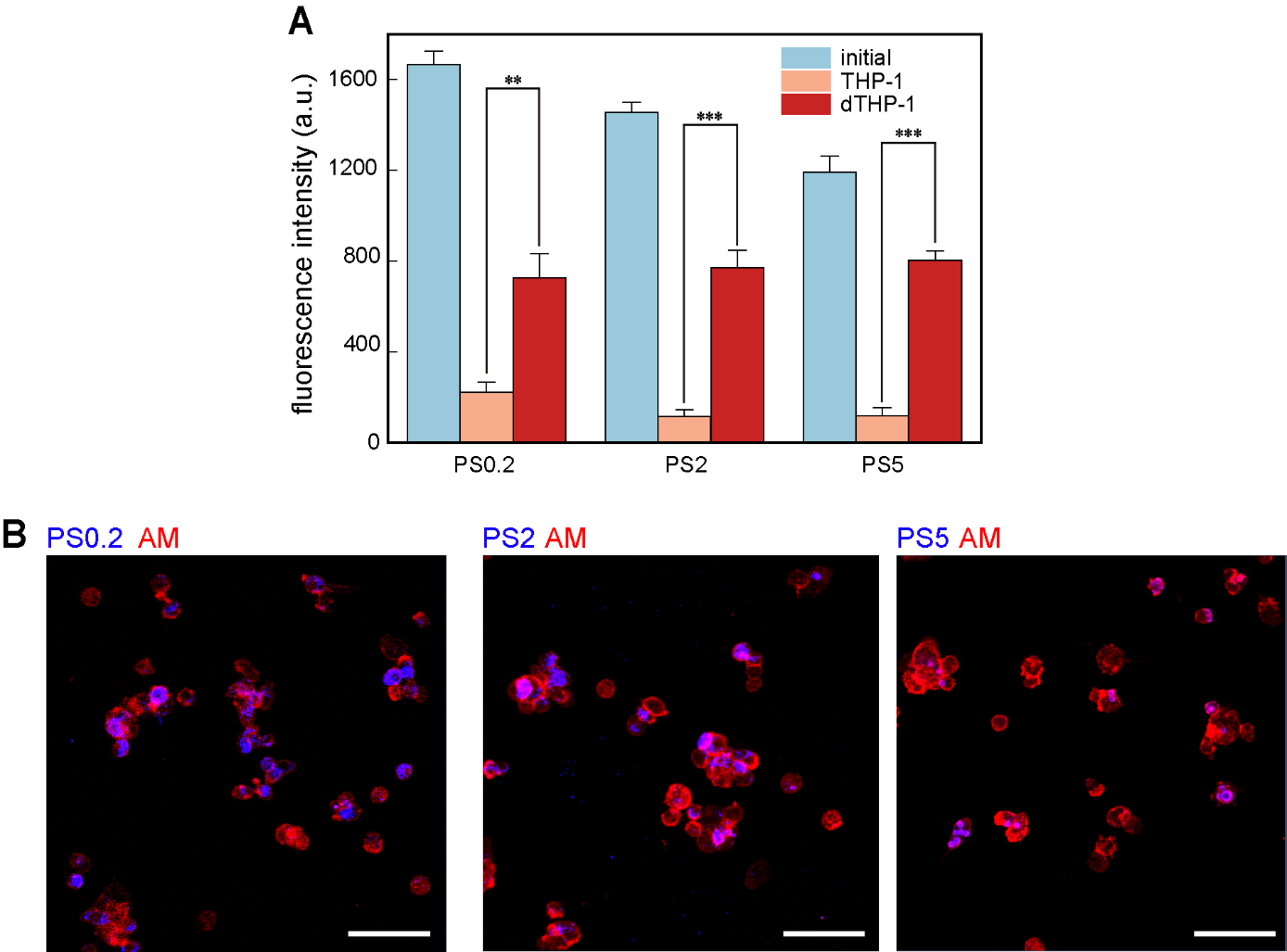


**Supplementary Figure S3**. Phagocytic uptake of PS particles by dTHP-1 cells. (A) Quantification of intracellular fluorescence intensity for PS0.2, PS2, and PS5 particles after 12-hour incubation with undifferentiated THP-1 cells (THP-1, beige) and differentiated THP-1 cells (dTHP-1, red). All particle types showed significantly higher uptake in dTHP-1 cells, indicating enhanced phagocytic capacity (*n* = 6 independent chambers; mean ± s.d.; ***p* < 0.01, ****p* < 0.01). (B) Representative confocal images showing PS particle internalization (blue) by dTHP-1 cells (red). Nuclei are stained with DAPI (purple). All particle sizes were observed within the cytoplasm of dTHP-1 cells (scale bars, 50 μm).


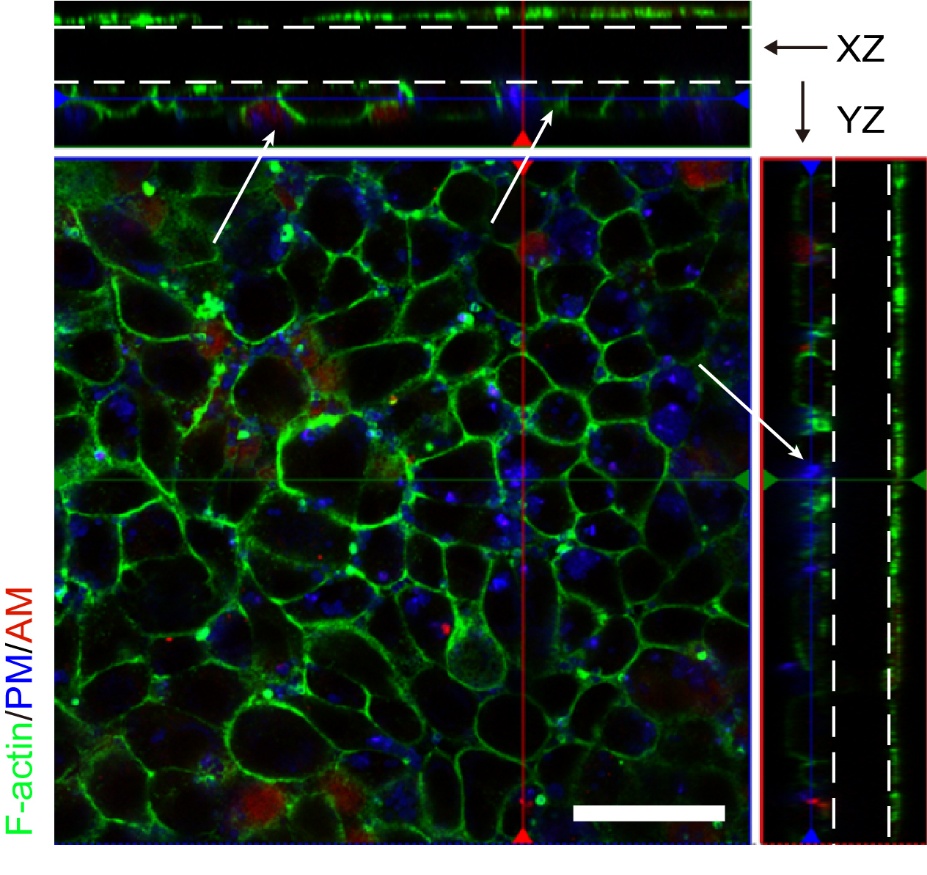


**Supplementary Figure S4**. Confocal visualization of PS2-loaded dTHP-1 cells in the ABB under low-intensity fluorescent staining. Confocal laser scanning microscopy (CLSM) image of the ABB model after addition of dTHP-1 cells. The epithelial layer is stained for F-actin (green), dTHP-1 cells are labeled with a red membrane dye, and PS2 particles are shown in blue. Orthogonal XZ and YZ projections reveal colocalization of blue particles within red-labeled dTHP-1 cells migrating across the epithelial layer. These observations confirm that dTHP-1 cells retain phagocytic activity and contribute to PS2 transport across the ABB (scale bars, 50 μm).


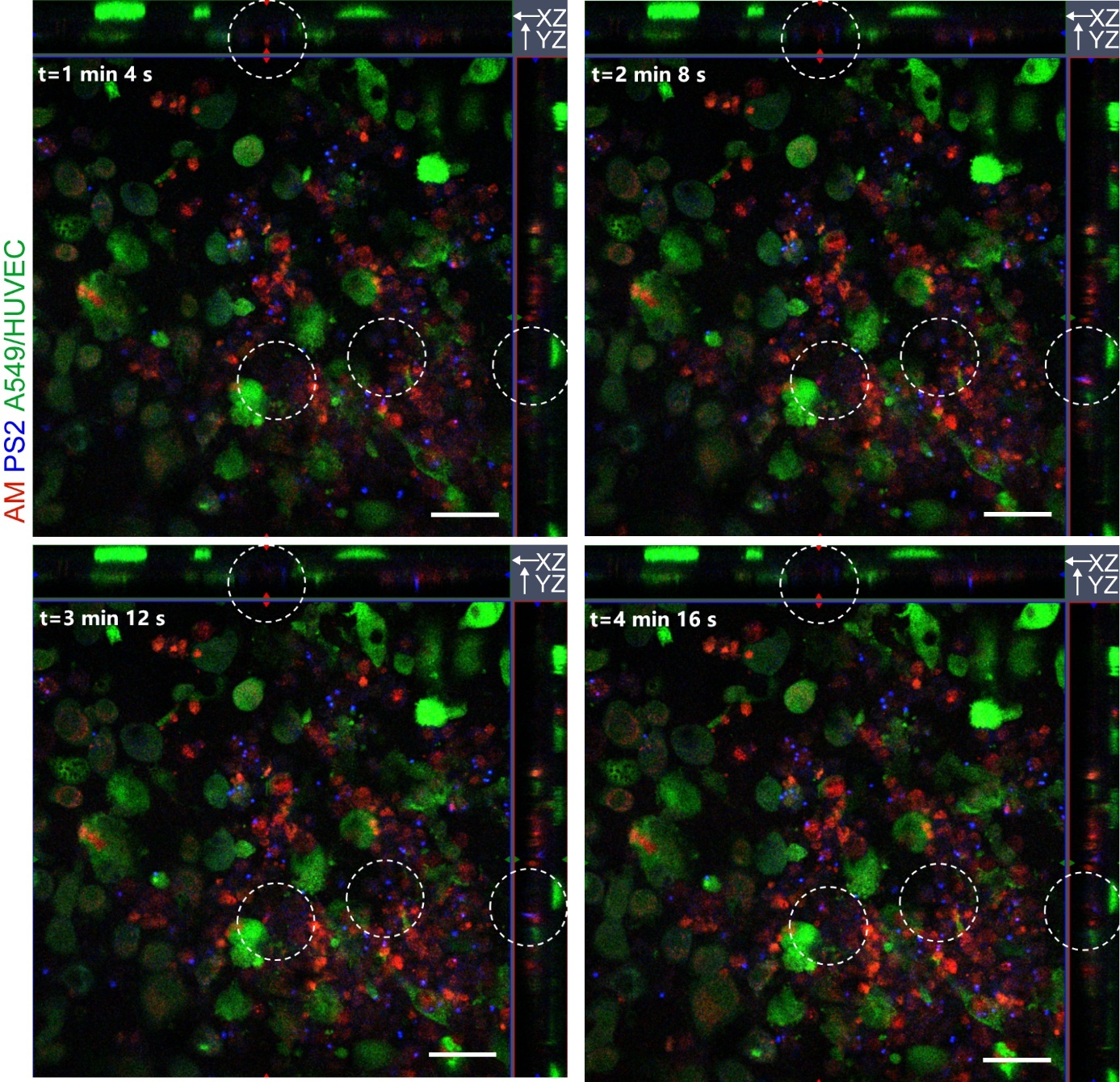


**Supplementary Figure S5**. **Macrophage carrying PS2 transmigrates across the air-blood barrier.** Time-lapse confocal images of a dTHP-1 macrophage (AM, red) with internalized 2 µm PS particles (PS2, blue) migrating through the epithelial/endothelial co-culture (A549/HUVEC, green). Dashed circles in the main and orthogonal (XZ, YZ) views highlight the vertical translocation of the macrophage (scale bars, 50 μm).

To provide direct visual evidence for this macrophage-mediated translocation, we performed live-cell time-lapse imaging of the process. As shown in the time-lapse series (**Figure S5** **Supplementary Video S2**), a dTHP-1 macrophage is observed migrating after having phagocytosed PS2 particles. The semi-transparent rendering of the macrophage (red) clearly reveals the internalized PS2 particles (blue) within its cytoplasm, confirming its role as a carrier.

The sequence tracks the movement of AMs, providing a dynamic view of the translocation process. Crucially, the orthogonal XZ and YZ projections, in conjunction with the main XY view, demonstrate a distinct vertical displacement of the macrophage. This confirms that the cell is actively transmigrating from the apical epithelial surface through the porous membrane, rather than simply moving laterally across the cell monolayer. This dynamic morphological evidence strongly supports our central finding that the translocation of 2 µm particles is actively driven by macrophage carriage, complementing the quantitative data presented elsewhere in the manuscript.


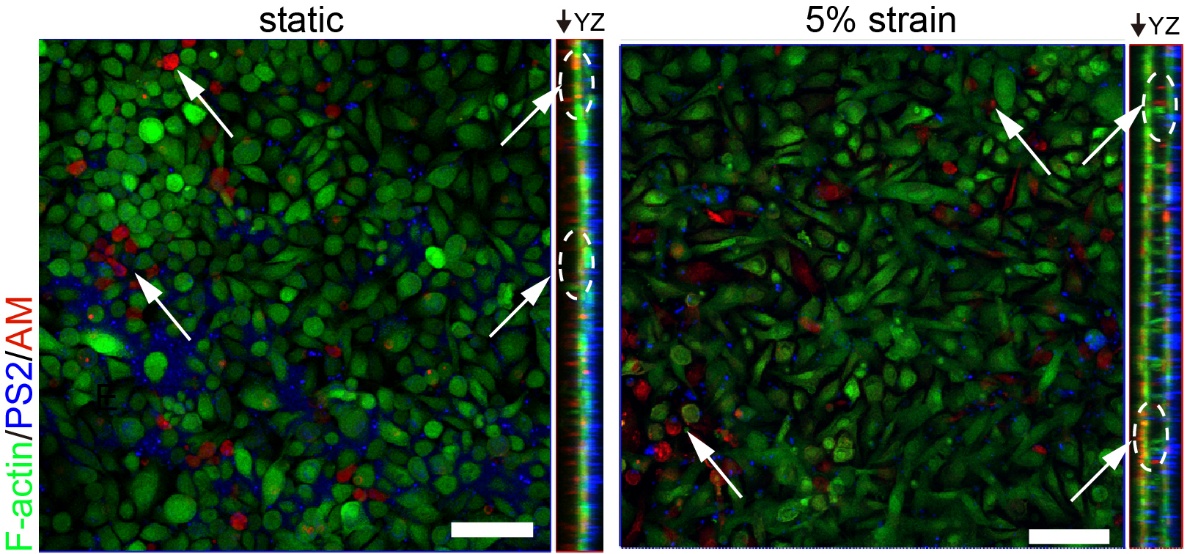


**Supplementary Figure S6**. Confocal visualization of PS2 particle uptake and translocation by dTHP-1 cells. Confocal laser scanning microscopy (CLSM) image of the ABB model after addition of dTHP-1 cells. The epithelial layer is stained for F-actin (green), dTHP-1 cells are labeled with a red membrane dye, and PS2 particles are shown in blue. Orthogonal XZ and YZ projections reveal colocalization of blue particles within red-labeled dTHP-1 cells migrating across the epithelial layer. These observations confirm that dTHP-1 cells retain phagocytic activity and contribute to PS2 transport across the ABB (scale bars, 50 μm).


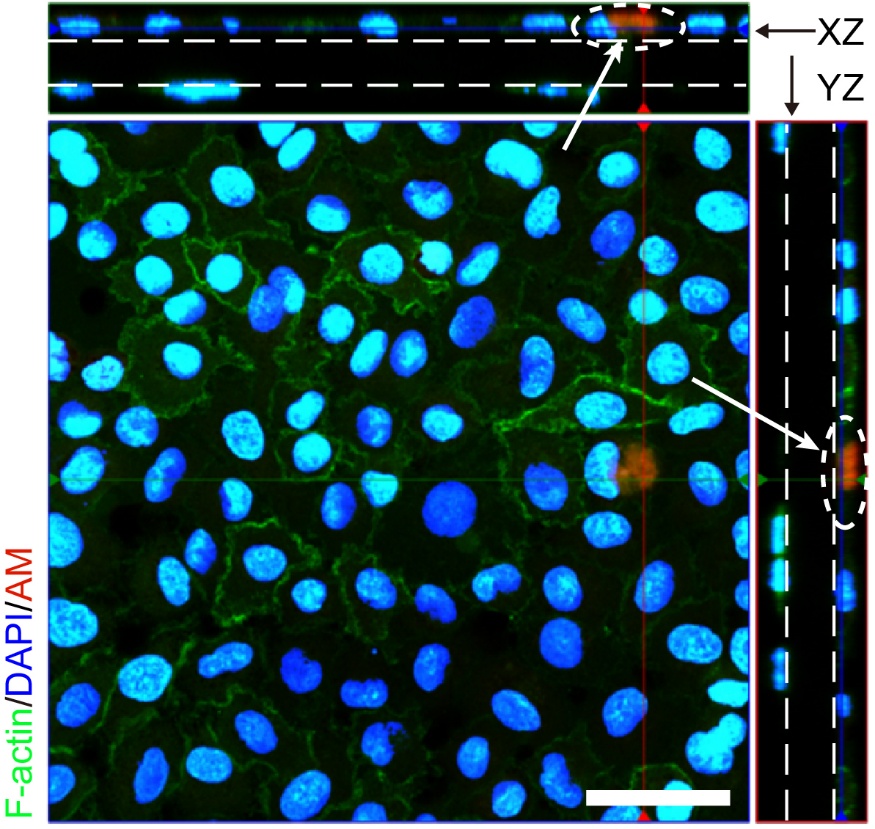


**Supplementary Figure S7**. Distribution of dTHP-1 cells in the ABB model without particle exposure. Confocal image showing dTHP-1 cells (red) in the ABB model under physiological conditions without PS particles. F-actin (green) outlines epithelial cells, and nuclei are stained with DAPI (blue). Orthogonal XZ and YZ projections show limited adhesion and vertical migration of dTHP-1 cells. These results indicate that without particulate stimulation, dTHP-1 cells remain mostly on the apical surface (scale bars, 50 μm).


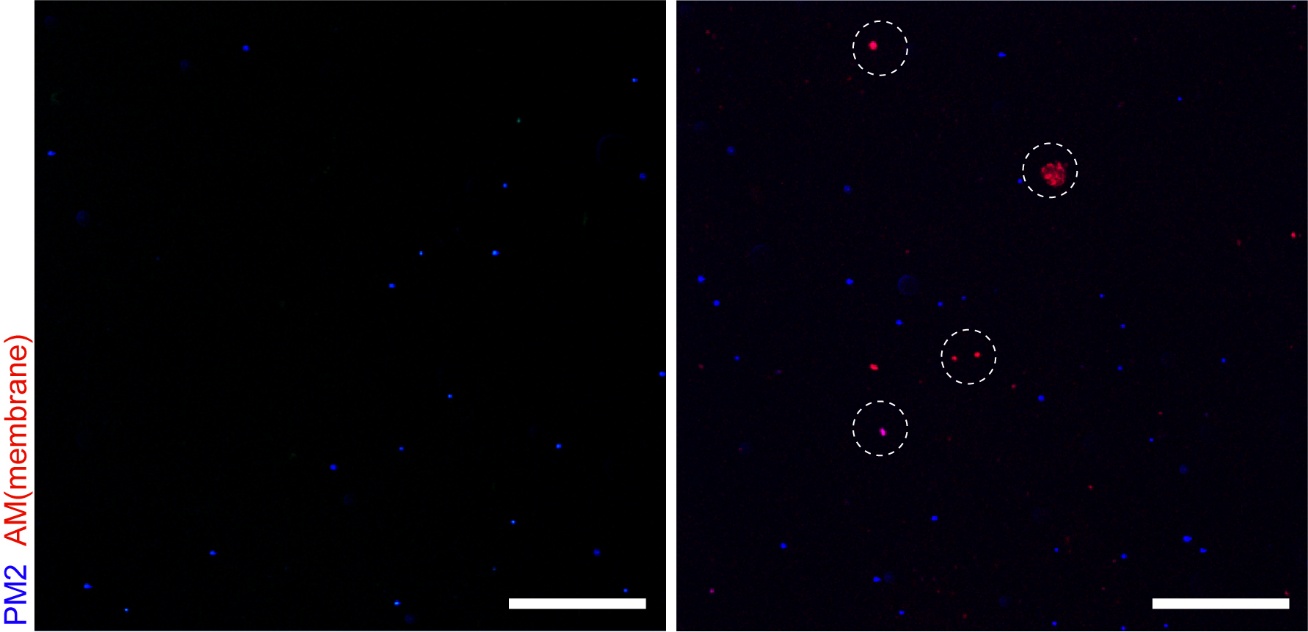


**Supplementary Figure S8.** PS2 particle distribution on the basal side of the ABB with or without dTHP-1 cells. Fluorescence images showing PS2 particles (blue) and dTHP-1 cell membranes (red) on the basal side of the ABB model. Left: Without dTHP-1 cells, PS2 particles appear sparse and scattered, indicating minimal passive translocation. Right: With dTHP-1 cells, dense PS2 clusters are observed, including regions colocalized with red macrophage membranes (circled), indicating active cell-mediated transport. These results confirm the essential role of macrophages in efficient translocation of micron-sized particles across the ABB (scale bars, 50 μm).


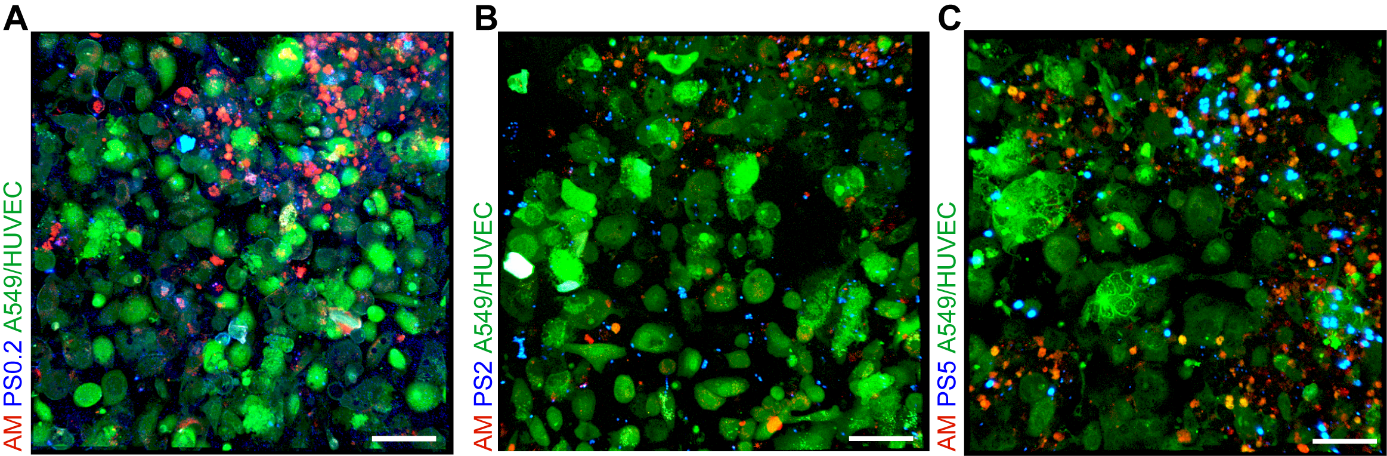


**Supplementary Figure S9. Initial seeding conditions of dTHP-1 cells within the ABB model prior to particle exposure.** Confocal microscopy images illustrate the cellular distribution of dTHP-1 cells (AM, red) co-cultured with A549/HUVEC cells (green) at a 3:1 seeding ratio. Images A, B, and C depict the cell density for experiments involving PS0.2, PS2, and PS5 particles respectively. In all conditions, particles were introduced at a concentration of 20 µg/mL (scale bars, 50 μm).

**Supplementary Video S1. Dynamic disruption of the air-blood barrier under cyclic strain.** The time-lapse video, captured using an IX83 fluorescence microscope, visualizes the response of the A549 epithelial monolayer (cytoskeleton stained red) to mechanical stress. The monolayer is first subjected to a physiological 5% cyclic strain and subsequently to a pathological 15% strain.

**Supplementary Video S2. Time-lapse imaging of macrophage-mediated translocation of a PS2 particle.** The confocal time-lapse video tracks a dTHP-1 macrophage (red) with a phagocytosed 2 µm PS particle (blue) as it transmigrates across the air-blood barrier formed by A549/HUVEC cells (green). The total imaging duration is 10 minutes and 40 seconds.

**Supplementary Video S3. Particle exocytosis from macrophages induced by 5 µM A23187.**

The confocal time-lapse video shows dTHP-1 cells laden with 2 µm PS particles (blue). The macrophage membrane is stained green, lysosomes are stained red, and brightfield images are included to visualize cell morphology and position. Following treatment with 5 µM A23187, a calcium ionophore, the active release of internalized particles is induced. The yellow box highlights a clear event of a particle being released with its associated lysosome, showing strong co-localization. A similar exocytosis trend is also observable in other cells in the field of view.

**Supplementary Video S4. Particle exocytosis from macrophages induced by 10 µM A23187.** The confocal time-lapse video shows dTHP-1 cells laden with 2 µm PS particles (blue), with the membrane stained green and lysosomes stained red. Brightfield images are included to observe cell morphology and position. Following treatment with a higher concentration of 10 µM A23187, a more rapid and pronounced release of internalized particles is observed. Yellow boxes 1 and 4 highlight particles that have been successfully exocytosed. Yellow boxes 2 and 3 show particles co-localized with lysosomes actively migrating towards the plasma membrane, exhibiting a clear trend towards imminent exocytosis.

**Reference**

[1] M. I. Hermanns, S. Fuchs, M. Bock, K. Wenzel, E. Mayer, K. Kehe, F. Bittinger, C. J. Kirkpatrick, *Cell and tissue research* **2009**, *336*, 91-105.

[2] K. J. Elbert, U. F. Schäfer, H.-J. Schäfers, K.-J. Kim, V. H. Lee, C.-M. Lehr, *Pharmaceutical research* **1999**, *16*, 601-608.

[3] D. Huh, B. D. Matthews, A. Mammoto, M. Montoya-Zavala, H. Y. Hsin, D. E. Ingber, *Science* **2010**, *328*, 1662-1668.

[4] J. D. Stucki, N. Hobi, A. Galimov, A. O. Stucki, N. Schneider-Daum, C.-M. Lehr, H. Huwer, M. Frick, M. Funke-Chambour, T. Geiser, *Scientific reports* **2018**, *8*, 14359.

[5] V. Suresh, *Cellular and Molecular Bioengineering* **2021**, *14*, 653-659.

[6] A. Costa, C. de Souza Carvalho-Wodarz, V. Seabra, B. Sarmento, C.-M. Lehr, *Acta biomaterialia* **2019**, *91*, 235-247.

[7] D. D. Nalayanda, C. Puleo, W. B. Fulton, L. M. Sharpe, T.-H. Wang, F. Abdullah, *Biomedical microdevices* **2009**, *11*, 1081.

[8] A. Fu, S. Mao, N. Kasai, H. Zhu, H. Zeng, *Biosensors and Bioelectronics* **2024**, *246*, 115858.

[9] N. Sevinc Ozdemir, S. Yaren Sahin, H. Kenar, V. Hasirci, *Nanomedicine* **2025**, 1-12.

[10] S. G. Klein, T. Serchi, L. Hoffmann, B. Blömeke, A. C. Gutleb, *Particle and fibre toxicology* **2013**, *10*, 31.
